# Proteomic View of Interactions of Shiga Toxin-Producing *Escherichia coli* with the Intestinal Environment in Gnotobiotic Piglets

**DOI:** 10.1371/journal.pone.0066462

**Published:** 2013-06-19

**Authors:** Rembert Pieper, Quanshun Zhang, David J. Clark, Prashanth P. Parmar, Hamid Alami, Moo-Jin Suh, Srilatha Kuntumalla, John C. Braisted, Shih-Ting Huang, Saul Tzipori

**Affiliations:** 1 J. Craig Venter Institute, Rockville, Maryland, United States of America; 2 Division of Infectious Diseases, Cummings School of Veterinary Medicine, Tufts University, North Grafton, Massachusetts, United States of America; Charité-University Medicine Berlin, Germany

## Abstract

**Background:**

Shiga toxin (Stx)-producing *Escherichia coli* cause severe intestinal infections involving colonization of epithelial Peyer’s patches and formation of attachment/effacement (A/E) lesions. These lesions trigger leukocyte infiltration followed by inflammation and intestinal hemorrhage. Systems biology, which explores the crosstalk of Stx-producing *Escherichia coli* with the *in vivo* host environment, may elucidate novel molecular pathogenesis aspects.

**Methodology/Principal Findings:**

Enterohemorrhagic *E. coli* strain 86–24 produces Shiga toxin-2 and belongs to the serotype O157:H7. Bacterial cells were scrapped from stationary phase cultures (the *in vitro* condition) and used to infect gnotobiotic piglets via intestinal lavage. Bacterial cells isolated from the piglets’ guts constituted the *in vivo* condition. Cell lysates were subjected to quantitative 2D gel and shotgun proteomic analyses, revealing metabolic shifts towards anaerobic energy generation, changes in carbon utilization, phosphate and ammonia starvation, and high activity of a glutamate decarboxylase acid resistance system *in vivo*. Increased abundance of pyridine nucleotide transhydrogenase (PntA and PntB) suggested *in vivo* shortage of intracellular NADPH. Abundance changes of proteins implicated in lipopolysaccharide biosynthesis (LpxC, ArnA, the predicted acyltransferase L7029) and outer membrane (OM) assembly (LptD, MlaA, MlaC) suggested bacterial cell surface modulation in response to activated host defenses. Indeed, there was evidence for interactions of innate immunity-associated proteins secreted into the intestines (GP340, REG3-γ, resistin, lithostathine, and trefoil factor 3) with the bacterial cell envelope.

**Significance:**

Proteomic analysis afforded insights into system-wide adaptations of strain 86–24 to a hostile intestinal milieu, including responses to limited nutrients and cofactor supplies, intracellular acidification, and reactive nitrogen and oxygen species-mediated stress. Protein and lipopolysaccharide compositions of the OM were altered. Enhanced expression of type III secretion system effectors correlated with a metabolic shift back to a more aerobic milieu *in vivo*. Apparent pathogen pattern recognition molecules from piglet intestinal secretions adhered strongly to the bacterial cell surface.

## Introduction

Enterohemorrhagic *E. coli* (EHEC) are a group of bacteria containing many serotypes responsible for outbreaks of bloody diarrhea occasionally leading to hemolytic uremic syndrome (HUS) and neurological abnormalities which in severe cases can be fatal [1]. These complications are attributed to Shiga toxins, one of which - Shiga toxin-1 (Stx-1) - is shared with *Shigella dysenteriae* type 1, acquired via horizontal gene transfer through phages [2]. The most common cause of outbreaks and sporadic cases of bloody diarrhea in the U.S. are strains of serotype O157:H7. EHEC strain 86–24 caused an outbreak of hemorrhagic colitis with a high mortality rate in 1986 [3] and produces only Shiga toxin-2 (Stx-2) [4]. Pig is the only species besides human naturally susceptible to the toxins’ systemic effects, and Stx-2 producers in particular have caused severe neurologic symptoms in the piglet model [5]. Although conventional piglets are naturally not susceptible to EHEC, we consider the gnotobiotic piglet model of infection with EHEC strains particularly useful to obtain systems-level insights into the molecular pathogenesis associated with Stx-production and with the characteristic bacterial attaching-effacing colonic lesions. which resembles that observed in humans [6].

The first genome of an *E. coli* serotype O157:H7 strain (EDL933) was published by Perna *et al.* [2]. A pathogenicity island in the genome was termed “locus of enterocyte effacement” (LEE). LEE-expressed proteins include the type III secretion system (T3SS) needle and translocator pore proteins (i.e., EspA, EspB and EspD), which fuse with the mammalian host cell membrane to allow the injection of effectors [7]. One LEE-encoded virulence factor is the transmembrane intimin receptor (Tir), which integrates into the host cell membrane and binds to the bacterial outer membrane (OM) protein intimin (Eae). This interaction triggers actin polymerization and pedestal formation, a pathological process characteristic of EHEC invasion of the colonic epithelium [7], [8]. The genes encoding the T3SS structural subunits, additional effectors manipulating host cell functions (e.g., EspF, EspG, and Map) and chaperones for the T3SS effectors also reside in the LEE gene cluster [7], [8]. The A/E lesions, which are formed following the epithelial cytoskeletal rearrangements, disrupt the boundary function of the mucosa, allow systemic release of Shiga toxins, and cause infiltration of inflammatory host cells. Nearly 40 potentially functional effectors not encoded by LEE were also identified [9] of which EspF_U_ [10] and NleH [11] were further characterized. StcE is a secreted metalloproteinase cleaving and inactivating host glycoproteins such as mucin-7 and glycoprotein 340 [12]. This enzyme is exported by a type II secretion system (T2SS) encoded by the virulence plasmid pO157. The transcription factor Ler regulates *stcE* and many of the LEE genes transcriptionally [13]. Protein abundance profiles of secreted effectors confirmed the important regulatory functions of Ler and the integration host factor Ihf [14]. Most of the effectors were absent in the secreted bacterial fraction of Δ*ler* and Δ*ihf* mutants of EHEC strain EDL933.

While the functional roles of many effectors and their transcriptional regulation *in vitro* and *in vivo* have been studied, far less information is available on system-wide adaptations of EHEC strains to their *in vivo* host environments. We reported the first comprehensive proteomic analysis of an EHEC strain (86–24) and identified more than 2500 proteins, but that study was not focused on environmental adaptation [15]. *In vivo* host adaptation surveys were previously performed for *Shigella dysenteriae* serotype 1 (SD1) using a similar gnotobiotic piglet model; acid resistance systems, anaerobic energy metabolism pathways and the expression of a T3SS system that mediates epithelial cell invasion were strongly induced in SD1 cells isolated from the piglet intestinal environment [16], [17]. Transcriptome analysis was applied to examine the adaptation of *E. coli* O157 strains to environments simulating *in vivo* conditions, such as the presence of red blood cell membranes [18] and the uptake by human macrophages [19]. Red blood cell membrane adherence resulted in higher gene expression for stress response regulators (*rpoE*, *rseB*, *phoQ*) and T3SS effectors (*eae*, *tir*, *cesT*, *map,* and *espA*), whereas the NtrC regulon responsible for nitrogen metabolism was strongly down-regulated in the examined *E. coli* O157 strains [18]. EHEC strain EDL933 revealed expression changes for 22% of all arrayed genes when residing in human macrophages compared to growth *in vitro* [19]. In addition, genes encoding iron transporters (*fit* and *chu*), acid resistance systems, and the Shiga toxin subunits *stx1B/2B* were increased in expression in the macrophage milieu, while the SOS response repressor *lexA* and several SOS response genes were down-regulated. Oxidative burst in macrophages is associated with increased production of reactive oxygen and nitrogen species. Gene expression levels for the peroxide stress-associated regulator *oxyR* and the oxidative stress response enzymes *katP*, *katE* and *katG* were surprisingly unchanged [19]. In a different study, decreased production of the signaling molecule indole and increased Curli biosynthesis were linked to enhanced biofilm formation in strain EDL933 compared to strain 86–24 [20].

The purpose of this study was to identify changes in the proteome of *E. coli* O157:H7, strain 86–24, resulting from physiological adaptations to piglet conditions that mimic hemorrhagic colitis in humans. Unlike macrophage and epithelial cell models, the gnotobiotic piglet model does not disregard the complexity and stages of infection as well as defensive responses by the host. We hypothesized that the analysis of proteome-wide changes allowed molecular insights into the pathogen’s survival and at least partial reconstruction of metabolic networks important *in vivo*. Data on proteomic host adaptations were previously reported for *Salmonella enterica Serovar Typhimurium* (*S. typhimurium*) [21], [22], and were extended towards the building of models of the pathogen’s *in vivo* metabolic capacity [23]. Here, we are not only taking steps into that direction, but also discovered innate immune response-associated proteins binding to the EHEC cell surface.

## Methods

### Ethics Statement

Experiments in piglets were performed to allow analysis of the *E. coli* O157:H7 proteome from *in vivo* environments. Piglets were delivered by caesarian section and housed at the Division of Infectious Disease of Tufts University School of Veterinary Medicine in accordance with approved procedures of the Institutional Animal Care and Use Committee at Tufts University. The animal studies were specifically approved by this committee. The respective protocols were No. G770-6, titled gnotobiotic piglet model of *E. coli* infection, date 4-24-2006, and No. G2009-52, titled gnotobiotic piglet model of *E. coli* infection, date 5-4-2009.

### Bacterial Cell Recovery from Cell Cultures and Animal Experiments

Cells of the *E. coli* O157:H7 strain 86–24 were isolated from *in vitro* cell cultures and *in vivo* animal experiments. Cells were grown *in vitro* in Luria-Bertani (LB) broth to stationary phase (OD_600_ ∼2.0) in a shaking incubator at 37°C, pelleted by centrifugation at 7,000×*g* for 10 min at 4°C, washed with a 20-fold volume of PBS and re-concentrated at 7,000×*g* for 15 min at 4°C. As for the animal experiments, several piglets developed diarrhea 18–30 h post-inoculation and were euthanized 3 to 5 days later. Bacterial cells (between 1×10^9^ and 2×10^10^ cells) were recovered from the piglets’ gut contents, repeatedly washed with PBS and purified via density gradient centrifugation with an isotonic 65% Percoll solution at 14,500×*g* for 30 min at 4°C, as previously described [17]. Up to 2×10^10^ bacterial cells were re-suspended either in 1 ml of TTE lysis buffer (25 mM Tris-OAc, pH 7.8, 0.05% Triton X-100, 5 mM Na-EDTA, and benzamidine and AEBSF in 1 mM concentrations) to process samples via shotgun proteomics or in 0.5 ml GR lysis buffer (8 M urea, 2 M thiourea, 4% (w/v) CHAPS, 18 mM DTT and 0.5% (v/v) Bio-Lyte pH 3-10 carrier ampholytes) for 2D gel analysis. 2D gel samples were frozen at -80°C until further use.

### Protein Extraction from Cell Lysates and Digestion

Samples to be subjected to shotgun proteomics were supplemented with chicken lysozyme (150 µg/ml) and agitated at 20°C for 1 h followed by quick-freezing and storage at −80°C until further use. To disintegrate cells further and solubilize proteins, sonication of the samples was followed by nucleic acid degradation (DNAse I and RNAse, at 5 µg/ml) and lysate agitation for 1 h at 20°C. The supernatant and insoluble pellet for each sample were separated by centrifugation at 16,100×g, and the pellet fraction re-extracted with a solution including 2.5 M NaBr. The supernatant was concentrated to 2 mg/ml protein and applied to an analytical SEC column (G3000-SWXL; 7.8 mm×30 cm; TOSOH Bioscience, USA). Proteins were chromatographically separated in PBS supplemented with 0.01% Triton X-100 into fractions representing the M_r_ segments >280 kDa, 280–80 kDa and 80–10 kDa (fractions termed F1-s_SEC_, F2-s_SEC_ and F3-s_SEC_, respectively). The insoluble pellet was termed F4-p. These four fractions containing roughly 60–100 µg protein were each subjected to digestion with trypsin using a method termed filter-aided sample preparation (FASP) [24]. Briefly, all four fractions for a given sample were concentrated in a Microcon YM-10 membrane filter unit (10 kDa M_r_ cut-off; Millipore, Billerica, MA). Twenty µl of a 1 M DTT stock solution and 12 µl of a 10% SDS stock solution were added to denature the proteins for 3 minutes at 95°C. Following alkylation, proteolytic digestions (trypsin/bacterial protein ratio of 1∶30 to 1∶50) were performed at 20°C overnight. Filtrates contained the peptide mixture collected by centrifugation at 14,000×*g* and rinsed three times with 500 mM and 50 mM NH_4_HCO_3_ solutions to recover the entire protein digestion mixture, and samples were lyophilized. Experimental details for all of the procedures presented here were published previously [15].

### Two-dimensional Electrophoresis and Differential Gel Display

EHEC lysates in GR buffer were thawed, incubated at 20°C for 30 min and vortexed intermittently to complete protein solubilization. Whole cell lysates were centrifuged at 16,100×g for 30 min, and supernatants were subjected to protein quantification using the 2D Quant Kit (GE Healthcare, Piscataway, NJ). Supernatant samples were subjected to 2D gel electrophoresis in batches of 12 gels using published procedures [25], [26]. Briefly, 1^st^ dimension protein analysis in 24 cm immobilized linear pH gradient strips (pH range 4–7; GE Healthcare) included gel rehydration loading of samples with ∼150 µg protein and electrophoresis for ∼60,000 Vh. Following reduction and alkylation steps, re-equilibrated strips were applied to 2^nd^ dimension SDS-PAGE slab gel electrophoresis (25×19.5×0.15 cm; 8–18%T) for ∼1,800 Vh. Gels were fixed, stained with Coomassie Brilliant Blue G250 (CBB), de-stained, subjected to gel image analysis (data acquisition as 16 bit TIFF images) and imported into the software tool Proteomweaver v4 (Bio-Rad, Hercules, CA). As described previously [25], [26], the gel image analysis proceeded with spot detection, matching, normalization, intensity averaging and spot annotation steps. The *in vitro* group consisted of eight gels, derived from three biological culture replicates. The *in vivo* group consisted of 15 gels, derived from four biological replicates (bacterial isolates were from the colon and, in one case, from the ileum of the infected piglets). The Mann-Whitney Test was used for statistical significance analysis of protein spot differences. It is a non-parametric two sample distribution-free t-test and assesses whether two independent samples of observations come from the same distribution. The p-values determined by this test were based on 8 (*in vitro* spot) and 15 (*in vivo* spot) intensity observations. Spot abundance ratios ≥1.5 with a p-value <0.05 were imported into the Multiple Experiment Viewer (MeV) software suite. Significant protein changes with an adjusted p-value <0.05 were recorded. The positions of 25 cytoplasmic protein spots were used as landmarks for M_r_ and pI calibrations in 2D gels. Analysis of the 500 most abundant spots resulted in high Mascot scores for *E. coli* EDL933 proteins suggesting lack of contamination with *Sus scrofa* (piglet) proteins.

### Two-dimensional LC-MS/MS Analysis and Database Searches

Protein digestion mixtures (four fractions for each bacterial lysate) were subjected to 1^st^ dimension peptide separation “off-line” on a polysulfoethyl-aspartamide column (4.6 mm×50 mm, Nest Group, USA). Strong cation exchange chromatography (SCX) was performed at a flow rate of 0.5 ml/min as follows: 100% buffer A (5 mM KH_2_PO_4_, 25% CH_3_CN, pH 3.0) for 10 min; linear gradient to 20% buffer B (5 mM KH_2_PO_4_, 0.7 M KCl, 25% CH_3_CN, pH 3.0) over 30 min; to 50% buffer B over 20 min; to 100% buffer B over 5 min; maintain flow at 100% B for 5 min. Nineteen fractions were collected, and some of the early- and late-eluting fractions were pooled due to low peptide content based on the UV trace at 214 nm. The final number of SCX fractions was 13 for each of the FASP digests. A total of 52 SCX fractions for one bacterial lysate were lyophilized and stored at −80°C. On the day of 2^nd^ dimension peptide separation (LC-MS/MS) using a nano-ESI LTQ linear ion trap mass spectrometer (Thermo-Fisher, San Jose, CA), the set of 52 fractions was re-suspended in 0.1% formic acid and transferred into a 96-well plate. In sequential, automated mode, a BioBasic C_18_ column (75 µm×10 cm; New Objective, Woburn, MA) was used for LC separations at a 350 nl/min flow rate [27]. The calibration was performed with 200 nmol human [Glu^1^]-fibrinopeptide B (M.W. 1570.57), verifying that elution times with a CH_3_CN gradient varied less than 10% and peaks representing ion counts had widths at half-height of <0.25 min, signal/noise ratios >200 and peak heights >10^7^. The loaded sample volume was 25 µl, and peptides were eluted from the C_18_ trapping cartridge into the C_18_ column with 53 min binary gradient runs from 97% solvent A (0.1% formic acid) to 80% solvent B (0.1% formic acid, 90% AcCN). Spectra were acquired in automated MS/MS mode, with the top five parent ions selected for fragmentation in scans of the m/z range 300–1,500 and a dynamic exclusion setting of 90 sec. LTQ search parameters (+1 to +3 ions) included mass error tolerances of ±1.4 Da for peptide precursor ions and ±0.5 Da for fragment ions (monoisotopic mass values), allowed one missed tryptic cleavage and were set for Cys carbamidomethyl modification and Met oxidation as fixed and variable modifications, respectively. The search engine used for peptide identifications was Mascot v2.3 (Matrix Science). The protein sequence databases derived from the genomes of *E. coli* O157:H7 strain EDL933 and *Sus scrofa* were downloaded from RefSeq in NCBI.

### Semi-quantitative Proteomic Analysis using Computationally Modified Spectral Counting

Protein identifications derived from Mascot v2.3 searches required at least one unique peptide with an e-value <0.1. Mascot search peptide false discovery rates (FDRs) were determined searching a randomized *E. coli* O157:H7 EDL933 decoy protein sequence database. The average FDR for 19 2D-LC-MS/MS experiments was 1.3%. Following file conversion into the mzXML format, MS data were re-scored using the algorithms PeptideProphet™ and ProteinProphet™ [28], [29]. Prot.xml files were analysed using an in house-developed software tool termed APEX quantitative proteomics tool v1.1 [30]. Using a pre-defined set of MS analysis parameters and 30 physicochemical properties provided by the APEX tool (default settings), ARFF files for *O_i_* computations were generated for a set of 100 highly abundant bacterial proteins with M_r_ values >30 kDa (according to 2D gel data) to achieve a good representation of tryptic peptides. Observation of tryptic peptides in the training dataset were correlated with the 30 physicochemical peptide properties, resulting in the computation of *O_i_* values (the expected number of unique proteotypic peptides for a given protein *i*) for all protein sequences, in this case 5397 annotated proteins in the *E. coli* O157:H7 EDL933 sequence database. The equation to calculate APEX_i_ values from 2D-LC-MS/MS data includes the O*_i_* correction factors, probability scores for protein identification (p_i_) and spectral counts (n_i_) as variables: 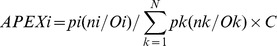 Setting the protein FDR at 1%, only proteins identified at a 99% confidence level were used for spectra counting. A factor of 2.5×10^6^, the estimated number of protein molecules per *E. coli* cell, was used to normalize APEX scores as protein molecules per cell [31].

### Bioinformatics and Statistical Methods

Spectral counting datasets analyzed by the APEX tool were imported into the software suite MeV (http://www.tm4.org/mev/) [32]. In MeV, 11 *in vitro* datasets (three biological replicates) and 7 *in vivo* datasets (from three piglets) were subjected to the non-parametric two sample Wilcoxon Rank Sum test to determine statistically significant abundance changes of proteins (p-value <0.05) comparing the two groups. The Benjamini-Hochberg procedure was applied for multiple testing corrections, calculating a FDR for each p-value [33]. The acceptable FDR for statistical significance of protein abundance changes was generally set at an adjusted p-value threshold of 0.05. Some protein changes were accepted to enhance biological data interpretations despite an adjusted p-value >0.05. A Wilcoxon Rank Sum was used to determine significant protein abundance ratios with a p-value <0.05 for the comparison of two distinct *in vivo* sample groups (the HB- and the HB+ groups, see Results; n = 4 and n = 3, respectively). Computational tools and literature evidence contributed to assignments of subcellular localizations, membrane attachment, molecular functions and pathway associations, as described previously [20]. Information on *E. coli* gene regulation, protein functional data and biochemical pathways was taken from EcoCyc and Uniprot (www.ecocyc.org and www.uniprot.org, respectively) to allow protein network analyses.

## Results and Discussion

Our study had three objectives. **First**, proteomic data from two sample groups were compared: EHEC cells cultured *in vitro* to mid-stationary phase versus those isolated post-mortem from the colon of gnotobiotic piglets inoculated with 5×10^9^ bacterial cells. This dose usually results in severe diarrhea (as compared to mild or moderate diarrhea at lower infectious doses), which invariably lead to systemic disease. Shotgun proteomic data consisted of 1,500 bacterial proteins on average, identified at a 1.3% peptide FDR using Mascot v2.3 [15]. Since APEX data estimate protein abundances as copies/cell, semi-quantitative comparisons of different proteins, e.g. those associated with a specific pathway, complex, or cellular localization, within a given sample group were possible. The differential 2D gel display of ∼500 identified protein spots provided relative abundances of proteins comparing the two sample groups. 2D gel profiles were less comprehensive, but very often confirmed the direction of a protein abundance change computed from the APEX datasets, as shown in Dataset S1 and the 2D gel maps in Figure S1. Proteins in the Dataset S1 are ordered according to functional categories, with annotation data for proteins and statistically significant protein abundance differences, if observed, for both APEX and 2D gel data. **Second**, we compared proteomic profiles of two piglet sample subgroups based on MS identification of *Sus scrofa* hemoglobin. The rationale was that hemoglobin was indicative of a more invasive stage of infection (Dataset S2). **Third**, predicting host protein-bacterial surface interactions, the *in vivo* MS data were searched with a *Sus scrofa* protein sequence database (Dataset S3). Data from cell cultures of strain 86–24, never in contact with host proteins, served to eliminate false positive protein hits such as trypsin, cytokeratin, and peptide sequences identical among EHEC and *Sus scrofa* protein orthologs. For the purpose of brevity, the terms “*increased, decreased or altered in vivo*” in the following paragraphs denote a protein abundance increase, decrease or change comparing *in vivo* and *in vitro* conditions, respectively. Quantitative data discussed in the following paragraphs pertain to APEX data, often included in Tables 1, 2 and 3, unless otherwise indicated.

**Table 1:** Selection of differentially abundant EHEC proteins involved in metabolic and transport activities.

Gene name	Protein description	*In vitro* average	*In vivo* average	In vitro/vivo ratio*	Trancriptional regulators ∧	Functional role $
aldA	lactaldehyde/glyceraldehyde dehydrogenase A, NAD-linked	6007	851	0.14	ArcA-, Fnr-, Crp+	aerobic EM: glycerol/sugar metabolism
glpK	glycerol kinase	32417	9134	0.28	Crp+, GlpR-	aerobic EM: glycerol/sugar metabolism
acnB	bifunctional aconitate hydratase 2/2-methylisocitrate dehydratase	10264	1789	0.17	ArcA-, FruR-, Fis-, Crp+	aerobic EM: TCA cycle
sdhA	succinate dehydrogenase flavoprotein subunit	14936	3130	0.21	Crp+,Fur+, Fnr-, Hfq-	aerobic EM: TCA cycle
icdA	isocitrate dehydrogenase	21055	4585	0.22	FruR+,ArcA-	aerobic EM: TCA cycle
aceA	isocitrate lyase	5878	501	0.09	FruR+,ArcA-, IhfB+	aerobic EM: TCA cycle, glyoxalate shunt
cyoA	cytochrome o ubiquinol oxidase SU2	1962	132	0.07	Crp+,ArcA-, Fnr-,FruR-	aerobic EM: electron transport chain
fbaB	fructose-bisphosphate aldolase	8277	1473	0.18	FruR-	aerobic EM: gluconeogenesis
argI	ornithine carbamoyltransferase SU1	40	1932	48.2	DksA+, ArgR-	amino acid BS: arginine
asnB	asparagine synthetase B	517	2452	4.74	GadX+	amino acid BS: asparagine
trpD	bifunct. anthranilate synthase/anthra- nilate phosphoribosyltransferase	1997	99	0.05	TrpR-	amino acid BS: chorismate/tryptophan
tnaA	tryptophanase	12198	2132	0.17	Crp+,TorR+	tryptophan metabolism and indole quorum sensing
serA	D-3-phosphoglycerate dehydrogenase	2161	6540	3.03	Crp+,Lrp+, leuLrp-	amino acid BS: serine
thrC	threonine synthase	1275	2985	2.34	Dks+	amino acid BS: threonine
dadA	D-amino acid dehydrogenase small SU	2209	64	0.03	Lrp-,leuLrp+	amino acid metabolism: alanine
astC	succinylornithine transaminase	1233	0	<0.01	NtrC+, ArgR+	amino acid metabolism: arginine
gcvT	glycine cleavage system aminomethyltransferase T	5099	1827	0.36	Lrp+,Fnr+, Crp+	amino acid metabolism: glycine
fsaA	fructose-6-phosphate aldolase	41	6786	163.7	–	carbohydrate metabolism: fructose
lacZ	galactoside O-acetyltransferase	59	18931	322.1	LacI-,HNS-	carbohydrate metabolism: galactose
galE	UDP-galactose-4-epimerase	633	7541	11.9	GalR+,HNS-	carbohydrate metabolism: galactose
gldA	glycerol dehydrogenase GldA	994	3433	3.45	HNS-	anaerobic carbohydrate metabolism: glycerol/glycol
L7018	putative cytochrome b562 family protein	120	755	6.31	–	anaerobic (?) EM: electron transport chain
yibO	phosphoglyceromutase	503	3673	7.31	FruR-	anaerobic (?) EM: glycolysis
ulaG	L-ascorbate 6-phosphate lactonase	0	2171	>100	UlaR+, IhfB-	anaerobic EM: ascorbate metabolism
dmsA	anaerobic dimethyl sulfoxide reductase SU A	14	589	41.2	Fnr+,NarL-, IhfB-, Fis-	anaerobic EM: electron transport chain
hybC	hydrogenase 2 large SU	511	3104	6.07	NarL-,ArcA-	anaerobic EM: electron transport chain
fdoG	formate dehydrogenase-O, major SU	813	1793	2.20	–	anaerobic EM: electron transport chain
nrfA	cytochrome c nitrite reductase	83	892	10.8	Fnr+,Fis-, NsrR-	anaerobic EM: electron transport chain, formate-dependent
adhE	bifunctional acetaldehyde-CoA/alcohol dehydrogenase	850	14975	17.6	Fnr+,FruR-, NarL-	anaerobic EM: mixed acid fermentation
fumB	fumarase B = fumarate hydratase class I; anaerobic isozyme	0	1921	>100	ArcA+,Fnr+, NarL-	anaerobic EM: mixed acid fermentation
yfiD	autonomous glycyl radical cofactor GrcA	4863	22467	4.62	ArcA+,Fis+, Fur-	anaerobic EM: mixed acid fermentation
acs	acetyl-CoA synthetase	4211	382	0.09	Crp+,Fis-	lipid and fatty acid biosynthesis
fabB	3-oxoacyl-(acyl carrier protein) synthase I	7565	3123	0.41	FadR+,FabR-	lipid and fatty acid biosynthesis
fadA	3-ketoacyl-CoA thiolase	2490	113	0.05	Fis+,ArcA-	lipid and fatty acid metabolism
amtB	ammonium transporter	0	2001	>100	NtrC+,GadX+	ammonia assimilation cycle
glnA	glutamine synthetase	2033	5807	2.86	Fis+	ammonia assimilation cycle
dcuA	anaerobic C4-dicarboxylate transporter	459	4172	9.08	Crp+,Fnr+, NarL-	nutrient/ion transport: dicarboxylic acids
fruA	fructose-specific PTS system IIBC SU	57	1407	24.6	FruR-	nutrient/ion transport: fructose-P
modA	molybdate transporter periplasmic protein	382	3249	8.50	Crp+,ModE-	nutrient/ion transport: molybdate
dppA	dipeptide transport protein	573	2015	3.52	IhfB+,Fnr-	nutrient/ion transport: peptides/amino acids
pstS	phosphate transport periplasmic SU	572	6824	11.9	PhoB+	nutrient/ion transport: phosphates
phoA	phosphate metabolism	9	1323	143.4	PhoB+	phosphate metabolism
fdx	[2Fe-2S] ferredoxin, electron carrier protein	2150	1084	0.5	-	redox: Fe-S cofactor
msrB	methionine sulfoxide reductase B	3335	374	0.11	Fis-	redox: methionine reduction
pntB	pyridine nucleotide transhydrogenase	912	3001	3.29	–	redox: NADH/NADPH conversion
pntA	NAD(P) transhydrogenase SU alpha	382	1307	3.42	–	redox: NADH/NADPH conversion
sthA	soluble pyridine nucleotide transhydrogenase	2484	260	0.1	–	redox: NADH/NADPH conversion

* *Differential abundance refers to in vivo versus in vitro protein abundance changes of the EHEC* proteins.

∧ Transcription factors involved in expression control of the respective genes are listed. +/−, positive/negative regulation.

$ (?) function during anaerobiosis inferred. Details are provided in Dataset S1.

**Table 2:** Selection of differentially abundant EHEC proteins involved in stress response and cell envelope structure.

Gene name	Protein description	*In vitro* average	*In vivo* average	In vitro/vivo ratio*	Trancriptional regulators ∧	Functional role $
yccZ	predicted exopolysaccharide export protein	411	1379	3.36	–	cell envelope: G4C capsular polysaccharide synthesis/export
lpxC	UDP-3-O-[3-hydroxymyristoyl] N-acetylglucosamine deacetylase	669	117	0.18	LexA-	cell envelope: lipid A core biosynthesis, lipopolysaccharides
lpxD	UDP-3-O-[3-hydroxymyristoyl] glucosamine N-acyltransferase	704	40	0.06	GadE+, CpxR+	cell envelope: lipid A core biosynthesis, lipopolysaccharides
L7029	putative lipid A biosynthesis (KDO)2-(lauroyl)-lipid IVA acyltransferase	8	668	88.0	–	cell envelope: lipid A core modification, lipopolysaccharides
arnA	UDP-L-Ara4N formyltransferase/UDP-GlcA C-4"-decarboxylase	1258	89	0.07	BasR+	cell envelope: lipid A core modification, lipopolysaccharides
wbpD	putative glycosyl transferase	621	230	0.37	–	cell envelope: O-antigen synthesis
galF	UTP–glucose-1-phosphate uridylyltransferase	1839	462	0.25	–	cell envelope: O-antigen synthesis
lolD	lipoprotein transporter ATP-binding subunit	498	0	<0.01	–	cell envelope: OM lipoprotein transport/assembly
bamA	Omp assembly factor YaeT	957	1928	2.02	(RpoE,CpxR)	cell envelope: OMP assembly
bamC	Omp assembly lipoprotein NlpB	1794	3324	1.85	(RpoE,CpxR)	cell envelope: OMP assembly
yajC	preprotein translocase SU YajC	2324	7083	3.05	–	cell envelope: protein secretion
secY	preprotein translocase SU SecY	480	1488	3.10	–	cell envelope: protein secretion
lsrB	autoinducer-2 binding protein lsrB	1917	0	<0.01	Lsr+,Crp+	chemotaxis and quorum sensing
cheY	chemotaxis regulatory protein CheY	837	0	<0.01	Fnr+	chemotaxis and quorum sensing
fliC	flagellin, filament structural protein	341	26	0.08	(CheY), GadE+	chemotaxis and motility
hmpA	nitric oxide dioxygenase	289	1352	4.68	MetR, Fnr-, NsrR-	detoxification: reactive nitrogen species
nirB	nitrite reductase (NADPH) large SU	0	830	>100	Fnr+,NarL+, FruR-,Fis-	detoxification: reactive nitrogen species
sodA	superoxide dismutase	3285	22	0.01	SoxS+,Fnr-, ArcA-	detoxification: reactive oxygen species
gstB	glutathione S-transferase B	1134	295	0.26	–	detoxification: xenobiotics
grxB	glutaredoxin 2	1781	306	0.17	–	Redox cofactor: reduction of oxidized proteins
phoP	DNA-binding transcriptional regulator PhoP	2671	984	0.37	(PhoP)	gene expression regulation: acid/low Mg adaptation
gadA	glutamate decarboxylase A	2.7 e6	5.5 e6	2.0*	ArcA+, GadX+	stress response: acid resistance
gadC	probable glutamate/gamma-aminobutyrate antiporter	559	1748	3.13	GadE+,GadX+Crp-,Fis-	stress response: acid resistance
yagU	inner membrane acid resistance protein YagU	436	1955	4.48	–	stress response: acid resistance
rstA	DNA-binding transcriptional regulator RstA	897	0	<0.01	PhoP+	gene expression regulation: low Mg adaptation
narL	transcriptional regulator NarL	1465	457	0.31	ModE+,Fnr-	gene expression regulation: nitrite-dep. anaerobic respiration
ompR	osmolarity response regulator	2085	368	0.18	IhfB-	gene expression regulation: environmental signals, osmolarity
osmC	osmotically inducible peroxidase	1483	75	0.05	RcsB+,HNS-	reactive oxygen/osmotic stress
ompC	outer membrane protein porin C	32645	53788	1.65*	OmpR+	controlled small molecule diffusion
iscR	DNA-binding transcriptional regulator IscR	525	45	0.09	(IscR)	gene expression regulation: iron-sulfur cofactor biosynthesis
glnK	nitrogen regulatory protein P-II 2	0	2706	>100	GadX+,NtrC+	gene expression regulation: response to nitrogen starvation
fis	factor for inversion stimulation, DNA-binding transcriptional dual regulator	0	1251	>100	IhfB+,DksA-	gene expression regulation: nucleoid structure and transcription
ihfB	integration host factor, SU beta	5197	1934	0.37	DksA-, (IhfA/IhfB)	gene expression regulation: nucleoid structure
lexA	LexA DNA-binding transcriptional repressor	460	18	0.04	(LexA)	gene expression regulation: SOS response, DNA damage
ygeY	predicted peptidase YgeY	3	3384	1229	–	proteolysis and peptidolysis (?)
ygeW	predicted catabolic transcarbamylase	0	910	>100	–	purine catabolism pathway (?)
eco	ecotin (trypsin inhibitor)	4220	307	0.07	–	proteolysis inhibitor (?)
hchC	chaperone protein HchA	3585	761	0.21	HNS-	stress response: protein (re)folding
cspA	DNA-binding transcriptional activator	0	5706	>100	Fnr-	cold shock and RNA chaperone
borW	putative Bor protein precursor of bacteriophage BP-933W	2307	9572	4.15	–	unknown function, located on mobile genetic element
yeeX	conserved protein YeeX	1424	4205	2.95	–	unknown function
Z2099	hypothetical protein Z2099	57	3951	69.1	–	unknown function

* Differential abundance refers to in vivo versus in vitro protein abundance changes of the EHEC proteins.

∧ Transcription factors involved in expression control of the respective genes are listed. +/−, positive/negative regulation.

$ (?) functional role not experimentally shown. Details are provided in Dataset S1.

**Table 3:** Selection of EHEC proteins differentially abundant in the hemoglobin-positive (HB+) group versus the hemoglobin-negative (HB-) in vivo group.

Gene name	Protein description	*In vitro* avg*	p-value in vivo/in vitro	*In vivo* ∧ (HB−)avg	*In vivo* ∧ (HB+)avg	Molecular functional roles
kdsB	3-deoxy-manno-octulosonate cytidylyltransferase	681	0.91	431	968	OM lipopolysaccharide biosynthesis, lipid A core
sopB	plasmid-partitioning protein SopB	902	0.077 ↓	86	797	Cell division, virulence plasmid O157 partitioning
minE	topolog. specificity cell division factor	2580	0.087 ↓	1012	2362	Cell division
espA	T3SS filament protein	206	0.73	43	1061	Forms T3SS needle structure
stcE	zinc metalloprotease	1863	0.15	150	1961	Cleaves mucin and gp340
eae	intimin receptor	386	0.75	156	2429	Receptor for intimate adhesion
cesT	T3SS effector chaperone CesT	1960	0.38	635	3128	Tir chaperone
tir	Intimin	821	0.59	480	2716	Translocated intimate adhesion effector protein
cesD	T3SS effector chaperone CesD	18	0.18	0	550	EspB and EspD chaperone
ihfA	integration host factor subunit alpha	4202	0.087 ↓	1738	3777	Global regulator influencing secretion of T3SS effectors
csrA	carbon storage regulator CsrA	1884	0.95	1025	3267	Involved in host invasion in *S. typhimurium*, glycogen storage
yhbH	sigma(54) modulation protein	682	0.88	181	1429	Modulates ribosomal activity
hfq	RNA-binding protein Hfq	7669	0.22 ↑	9135	16288	Post-transcriptional regulation of genes via srRNA binding
slp	carbon starvation lipoprotein	1676	0.144 ↑	3830	1695	Carbon starvation
ibpA	heat shock chaperone IbpA	341	0.91	0	2290	Protein aggregation stress
ibpB	heat shock chaperone IbpB	0	0.077 ↑	0	943	Protein aggregation stress
msrB	methionine sulfoxide reductase B	3335	0.007 ↓	210	595	Response to oxidative stress
nirB	nitrite reductase, NAD(P)H subunit	0	0.007 ↑	1228	299	Nitric oxide detoxification
yiiS	Conserved protein YiiS	1994	0.75	4283	1373	RNS-regulated gene in ETEC; RNS controls pilus expression
mdtE	multidrug resistance efflux protein	143	0.38	687	31	Efflux of toxins, antimicrobials and detergents
mdtF	multidrug resistance efflux protein	37	0.25	344	0	Efflux of toxins, antimicrobials and detergents
mscS	anion selective mechanosensitive channel MscS	525	0.004 ↑	2139	761	Small anion/water transport, response to mechanical stress
bioB	biotin synthase	0	0.077 ↑	0	462	Biotin cofactor synthesis

∧ Average (avg) abundance changes derived from HB+ and HB- in vivo sample groups (p<0.05) were also compared with the in vitro protein abundance data (*). The statistical significance of the in vivo vs. the in vitro change and an observed increase ↑or decrease ↓ are also provided. Details are provided in Dataset S2.

### EHEC Metabolism, Homeostasis and Nutrient Acquisition

**Figure 1:**
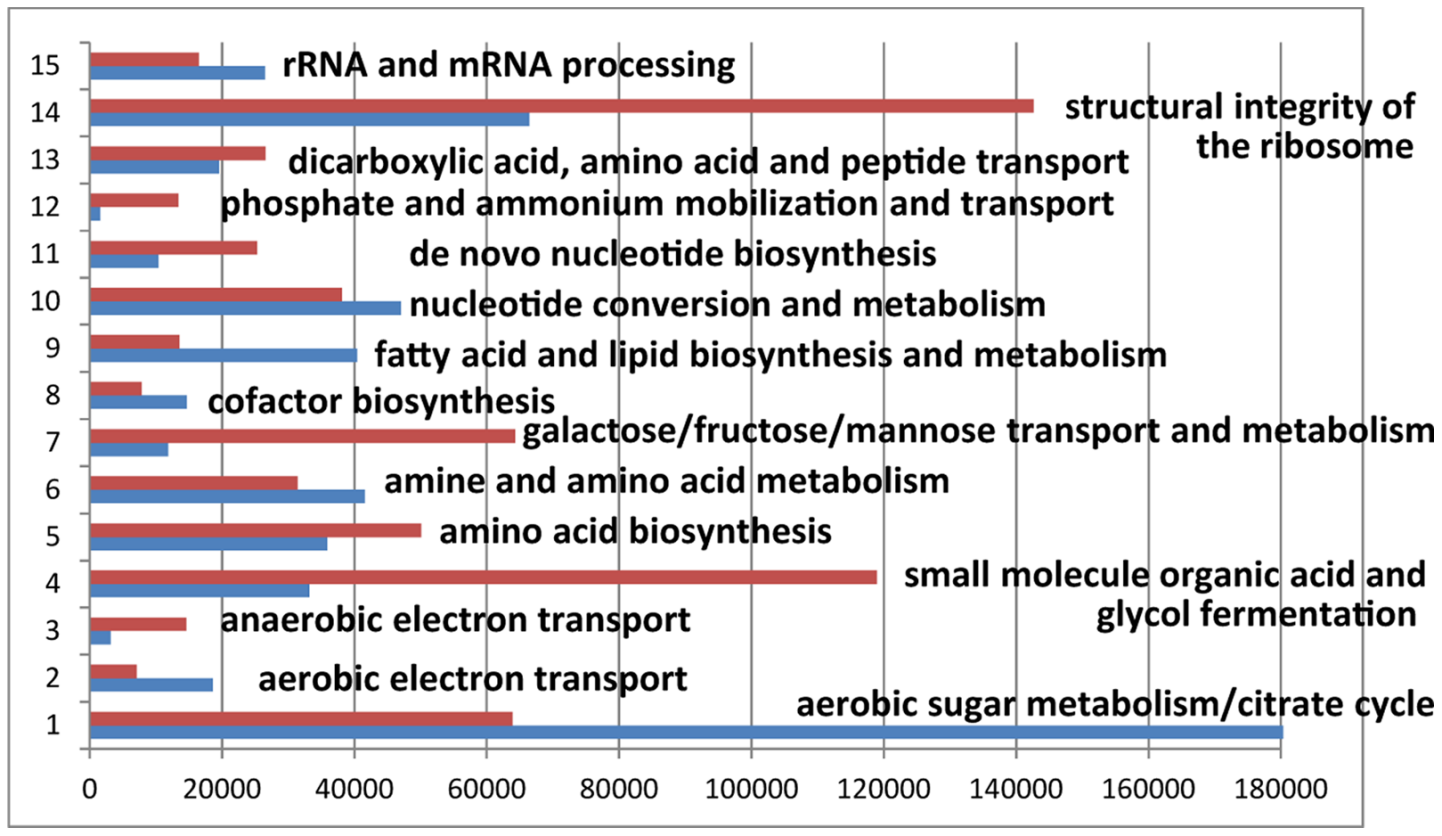
Global adaptation of EHEC cells to the intestinal milieu. Fifteen biological role categories, as defined in Dataset S1, are displayed in the graph. The bar length represents the sum of APEX_i_ quantities of all proteins with a statistically significant abundance change (*in vitro* versus *in vivo*) assigned to a given biological role category. Blue bars represent the *in vitro* (cell culture) growth, red bars the *in vivo* (intestinal) environments.

**Figure 2:**
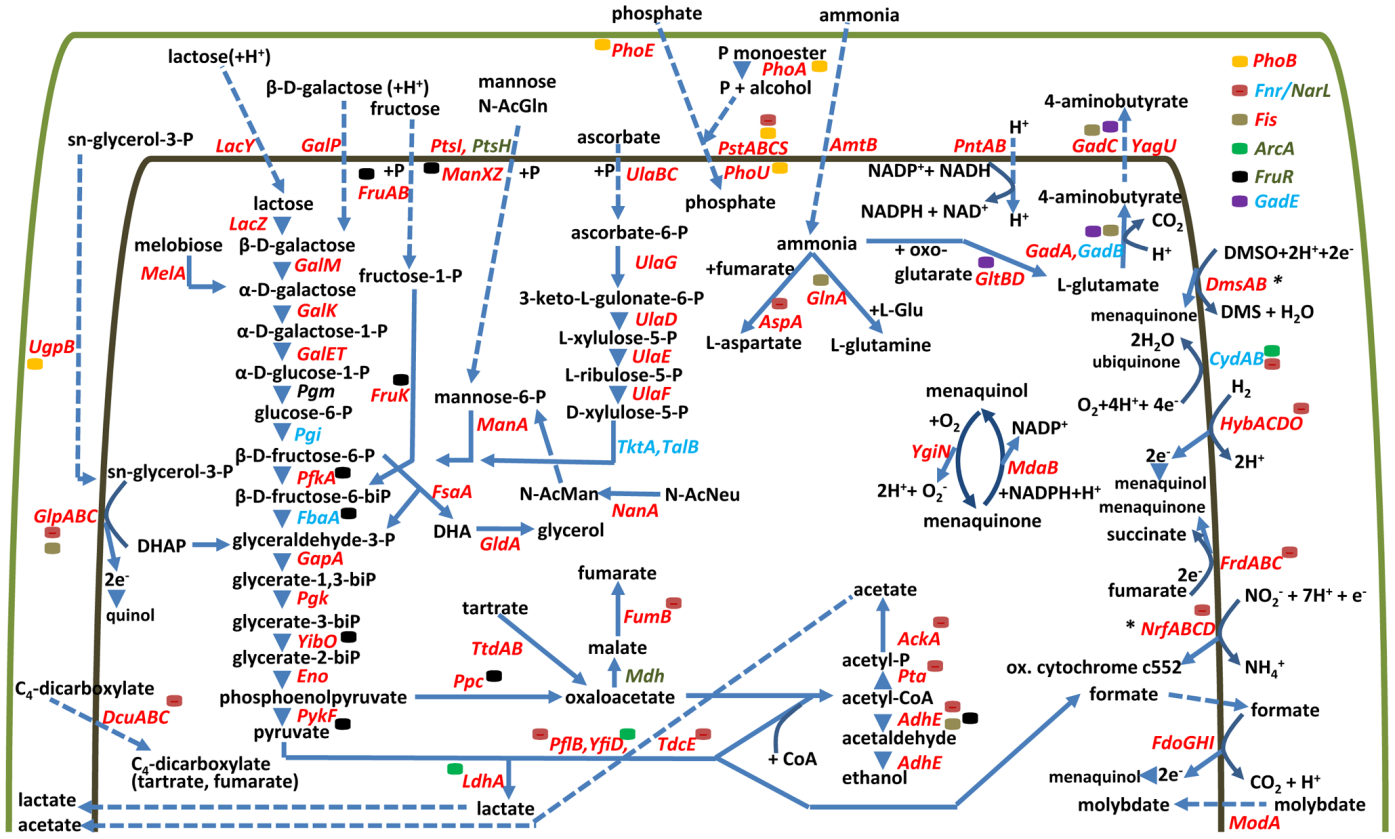
Metabolic pathways active in intestinal EHEC cells. From the left to the right: import and metabolism of glycerol derivatives and dicarboxylic acids; import of mono- and disaccharides and their entry into the glycolytic pathway (GP); metabolism of ascorbic acid; mixed acid fermentation utilizing GP products; high affinity phosphate and ammonia import and metabolism; NADPH generation and menaquinone/menaquinol cycle; glutamate decarboxylase acid resistance system (Gad); oxygen-dependent and -independent electron transport chains; *periplasmic electron transporters. Symbols/color codes: substrates and products of pathways are denoted in black script, proteins in red script (if increased in abundance *in vivo*), in blue script (if there was no abundance change but high expression *in vitro* and *in vivo*), in green script (if decreased in abundance *in vivo*); full arrows indicate a catalytic step, dotted arrows a transport step. In the top right corner, seven global transcriptional regulators are depicted; their color-coded symbols are included in the graphic to illustrate where they influence gene expression. The area between the green line (outer membrane) and inner line (inner membrane) represents the periplasmic space.

Figure 1 depicts fifteen broad biological role categories which, based on summed abundances of proteins assigned to the categories, revealed quantitative differences comparing the *in vitro* and *in vivo* conditions. Marked changes were related to ribosome structural integrity; phosphate and ammonium mobilization and transport; *de novo* nucleotide biosynthesis; galactose, fructose, and mannose transport and metabolism; fatty acid lipid biosynthesis and metabolism; small organic acid and glycerol fermentation; aerobic carbon metabolism and TCA cycle; aerobic electron transport; and anaerobic electron transport. The schematic in Figure 2 serves to establish pathway links among the proteins assigned to these categories, with a focus on molecular processes highly active in EHEC cells isolated from the piglets’ guts. The import and metabolism of several sugars were activated *in vivo*, consistent with carbon resources included in the piglets’ diet. Lactose, fructose, and mannose were taken up, metabolized and fed into the glycolytic pathway (GP) at higher rates *in vivo*. Transporters for 2-deoxy-D-galactose (GalP) and lactose (LacY) were expressed only *in vivo*; β-galactosidase (LacZ) was increased 322-fold *in vivo* (Table 1). Enzymes converting β-D-galactose to α-D-glucose-1-phosphate (GalM, GalK, GalE, GalT) and MelA whose product is α-D-galactose were also increased *in vivo*. FruA and FruB, responsible for fructose transport, were 24-fold more abundant *in vivo*. FruK, the enzyme phosphorylating β-D-fructose-1-phosphate prior to GP entry [34], and phosphofructokinase (PfkA), the main regulatory GP enzyme, were strongly increased *in vivo*. The class I D-fructose-6-phosphate aldolase FsaA revealed a 163-fold abundance increase *in vivo* (Table 1). Its biological pathway associations are not entirely understood [35]. We assume that FsaA has a metabolic bypass function when D-fructose-6-phosphate accumulates as a result of insufficient acetyl-CoA turnover in the citric acid cycle. This notion is supported by a concurrent 3.5-fold *in vivo* abundance increase of GldA (Table 1), the enzyme that reduces one of the two end-products of FsaA, dihydroxyacetone, to glycerol [36] (Figure 2). Also consistent with this conclusion were decreased *in vivo* quantities of all citric acid cycle and glyoxalate bypass enzymes. FsaA was previously reported to be active at low phosphate concentrations in *E. coli* [35]. As described later, there was strong evidence of a limited phosphate supply in intestinal EHEC cells.

Catabolic enzymes and transporters for C_2_- to C_6_-organic acids and glycerol derivatives were more abundant *in vivo*, suggesting bacterial responses to high quantities of the acids in the piglet intestine. The periplasmic subunit of the sn-glycerol-3-phosphate ABC transporter (UgpB) was increased *in vivo*. This C_3_-carbon intermediate may result from the turnover of bacterial or host phospholipids *in vivo*. Via oxidation to dihydroxyacetone-phosphate (DHAP), the molecule enters the GP as an energy source. At low oxygen concentrations, GlpA/GlpB/GlpC, a membrane-bound enzyme complex also increased in abundance *in vivo*, catalyzes sn-glycerol-3-phosphate oxidation [37] (Figure 2). The C_4_-dicarboxylate transporters DcuA and DcuB were increased in abundance more than 10-fold *in vivo*; their substrates include tartrate and fumarate [38]. Consistent with increased import activities for these acids, tartrate dehydrogenase (TtdA/TtdB) and fumarase B (FumB) (Table 1) were detected only *in vivo*. High activity of the enzymes have been linked to anaerobic bacterial biofilm formation [39]. Oxygen concentrations are generally low in the intestinal lumen. The ability to form a strong biofilm was considered a key virulence attribute of the EAEC O104:H4 strain that caused an epidemic systemic disease outbreak three years ago [40]. Proteins facilitating ascorbate uptake and catabolism also revealed 4 to 6-fold abundance increases *in vivo* (Figure 2). The product of the Ula pathway, D-xylulose-5-phosphate, is fed into the GP via the pentose phosphate pathway [41]. Transcriptional regulators involved in activating short organic acid metabolism were also profiled. FruR, the catabolite repressor activator, was 2.2-fold decreased *in vivo*. FruR represses many of the genes/operons encoding aforementioned transporters and enzymes [42] (Figure 2), and its de-repression appears to be a critical factor in redirecting bacterial carbon flow in the intestinal milieu.

There was strong evidence for phosphate and ammonia starvation in EHEC cells *in vivo*. In addition to UgpB which contributes to phosphate mobilization, the anion-specific outer membrane porin PhoE, subunits of the high affinity phosphate transporter Pst, periplasmic alkaline phosphatase (PhoA) and the auxiliary factor PhoU were strongly induced *in vivo* (Table 1/Figure 2). These proteins are part of the Pho regulon controlled by the two-component signal transduction system (2-CST) PhoB/PhoR [43]. Both the response regulator (PhoB) and the sensor histidine kinase (PhoR) were only detected in bacterial cells from the intestine. Several observations were indicative of strong ammonia mobilization in EHEC cells *in vivo*, supporting the biosynthesis of compounds essential for bacterial growth such as peptides, proteins and nucleotides. Aspartate-ammonia lyase was highly abundant *in vivo*. Subunits of the ethanolamine ammonia-lyase complex (EutL/EutC/EutB/EutE/EutM) were detected only *in vivo*. The inner membrane NH_4_^+^ transporter AmtB, highly increased in bacterial cells from the intestine (Table 1), facilitates ammonia import followed by cytoplasmic NH_4_^+^ assimilation via L-glutamine synthetase (GlnA) as shown in Figure 2 [44]; GlnA was also increased nearly 3-fold *in vivo* (Table 1). In contrast, enzymes with catalytic roles in amino acid metabolism (DadA, DadX, GcvP/GcvT, SdaA, Tdh, and Kbl) were strongly decreased *in vivo*. The nitrogen-regulatory protein PII-2 (GlnK) interacts with AmtB after ammonia shock [45]. GlnK (Table 2) and a 2-CST implicated in the response to nitrogen starvation (GlnG/GlnL) [46] were identified only in the intestinal EHEC proteome. In summary, the bacterial cells metabolically adjusted to increased availability of C_2_- to C_6_-organic acids and decreased availability of important nutrients such as PO_4_^3−^ and NH_4_^+^ *in vivo*.

There was evidence for a re-directed EHEC energy metabolism as a consequence of microaerophilic conditions in the pig intestinal lumen. Citric acid cycle enzymes (Table 1) and NADH dehydrogenase (Nuo), the main *E. coli* membrane complex for aerobic respiration, were decreased strongly and moderately, respectively. Two global regulators sensing O_2_ concentrations in bacterial cells (ArcA/ArcB and Fnr) [47] were identified (Figure 2). Unexpectedly, ArcA was decreased 2-fold in abundance *in vivo*. Post-translational events modulate activities of 2-CST response regulators, and their abundances do not necessarily correlate with their activities. Enzymes positively regulated transcriptionally by ArcA, and particularly Fnr, were increased *in vivo* (Table 1/Figure 2). Some of the enzymes catalyze the catabolism of two glycolytic products, pyruvate and phosphoenolpyruvate. Branches supporting the break-down of these metabolites are designated mixed acid fermentation (MAF) [48]. The fermentation products are lactate (activity of LdhA), acetate (activities of Pta and AckA), formate (activity of formate dehydrogenase-O; FdoG/FdoH/FdoI), fumarate (activity of fumarate reductase; FrdA/FrdB/FrdC/FrdD) and ethanol (activity of AdhE) (Figure 2). Additional enzymes metabolizing MAF intermediates (Ppc, PykF, PflB, TdcE, AdhE, FumB, and YfiD) were also invariably increased *in vivo*. AdhE, PflB and YfiD were among the *in vivo* most abundant cytoplasmic proteins of strain 86-24 (Table 1). High abundance of cytochrome *bd* terminal oxidase (CydAB), an oxidoreductive complex that depends on O_2_ as electron acceptor, suggested that the EHEC-colonized environment was microaerophilic rather than anaerobic [49]. Major electron donors for O_2_-independent electron transport *in vivo* were formate dehydrogenase-O and hydrogenase-2 (HybA/HybC/HybD/HybO) whose subunits (e.g., FdoG and HybC, Table 1) were increased *in vivo*. Electron acceptors contributing to O_2_-independent electron transport were fumarate reductase (Frd) and formate-dependent nitrite reductase (NrfA/NrfB/NrfC/NrfD). Both Hyb and Nrf are oxidoreductases localized at the *E. coli* inner membrane-periplasmic interface [50], [51]. Activity of the Fdo complex is coupled to Nrf and dimethylsulfoxide reductase (DmsA/DmsB), another membrane-associated electron carrier elevated in abundance *in vivo*. As depicted in the schematic of Figure 2, these redox complexes generate an electrochemical gradient across the inner membranes and a proton motive force, resulting in ATP production. A nine-fold increase of the periplasmic subunit of molybdate ABC transporter ModA was measured *in vivo* (Table 1). Molybdate is a DmsA cofactor [52]. Its import may be needed for Dms function in EHEC cells. Fnr regulates gene expression for some of the MAF branches and electron transporters indirectly via NarL, a transcription factor active at elevated nitrate or nitrite concentrations. NarL induces expression of *nuo* and nitrate reductase genes, whereas it represses *pflB*, *adhE*, *hybACDO*, *fumB*, and *dmsA/dmsB* expression [53]. A three-fold decrease of abundance of NarL was detected *in vivo* (Table 2), and de-repression of NarL-controlled genes *in vivo* can be inferred from numerous abundance changes, including five such proteins listed in Table 1.

Marked abundance differences were detected for two *E. coli* pyridine nucleotide transhydrogenases. Cytoplasmic transhydrogenase SthA specialized on re-oxidation of NADPH [54] was 10-fold decreased*;* membrane-bound PntA/PntB increased 3.4-fold *in vivo* (Table 1). PntA/PntB activities are associated with a balanced intracellular supply of NADPH for various biosynthetic processes [54]. Biosynthesis of amino acids (Table 1) and nucleotides *de novo*, each requiring NADPH in several catalytic steps, was indeed activated *in vivo*. In agreement with this data, the NADPH-consuming menaquinone-menaquinol redox cycle [55] also seemed to be mobilized in EHEC cells *in vivo*, judged from 1.7- and 2.5-fold increases of the enzymes MdaB and YgiN, respectively (Figure 2).

### EHEC Responding to Stress Derived from the Intestinal Environment

During the distinct infectious stages, the piglet’s defensive responses likely challenge EHEC stress response systems in different ways. Bacterial cells are exposed to various local secretion products in the gastro-intestinal tract. There is *ex vivo* and *in vivo* evidence for interactions of EHEC cells with specialized microfold (M) cells in Peyer’s Patches of the colon, contributing to localized innate immune defenses [56]. Penetration of the mucosa would expose the bacteria to erythrocytes, direct attacks by phagocytes, release of oxidative stressors and toxins, and the adaptive immune system. Here, the stress responses are examined as “averages” over distinct infectious stages (we isolated EHEC cells from the colon and ileum, with few proteomic differences); *in vitro vs. in vivo* comparisons lose some spatial and temporal resolution.

### Osmotic Stress

Ten osmotic stress response proteins were quantitatively changed in abundance nine of which were decreased considerably in intestinal *vs*. *in vitro* (stationary phase) bacterial cells. This included OsmC (Table 2), OsmY, YjbJ, YbaY and entericidin B (EcnB). EcnB, 3-fold decreased *in vivo*, is a bacteriolysin and causes cell death at high osmolarity, a process inhibited by the lipopeptide EcnA and a toxin-antitoxin binding mechanism [57]. EcnA was neither detected *in vivo* nor *in vitro*. EcnB expression is repressed by OmpR, a global cell envelope stress regulator. However, OmpR was 3-fold decreased *in vivo*. Without data on its phosphorylation, fewer insights in transcriptional control activities are obtained. OmpR also controls the expression of OmpC and OmpF, trimeric porins facilitating small hydrophilic solute diffusion across the OM [58]. OmpC was highly abundant in the EHEC proteome *in vivo* (Table 2), whereas OmpF was not detected. In the intestinal milieu, a high OmpC/OmpF ratio may limit diffusion of toxic molecules including colicins, bile acids and antimicrobial peptides into bacterial cells because OmpF has a larger pore size. Indeed, phosphorylated OmpR induces *ompC* expression, lending support to the notion of high *in vivo* OmpR activity [59]. MscS, a mechanosensitive channel involved in water homeostasis and part of the RpoS regulon, was increased 3-fold *in vivo* (Table 3). MscS has been linked to osmotic down-shock responses [60], supporting osmotic down-shock in EHEC cells exposed to the intestinal environment.

### Protease-induced Stress

EHEC cells encounter secreted proteases (pancreatic and intestinal) passing through the mammalian gut. Since the OM is a porous barrier towards the cells’ exterior, these insults implicate proteolytic damage in the bacterial cell envelope. There was little evidence for elevated abundances of chaperone-protease systems (e.g. PpiD, DegQ, HtrA, and SurA), which remove misfolded and aggregated proteins in the periplasmic space *in vivo*. Ecotin and the chaperone HchA were 14-fold and 5-fold decreased, respectively, *in vivo* (Table 2). *E. coli* HchA is part of the RpoS regulon and responds to osmotic up-shock [61]. The function of ecotin as a trypsin inhibitor [62] in the bacterial periplasm must be questioned considering the exposure of EHEC *in vivo* to secreted trypsin. A predicted peptidase (YgeY) and a transcarbamylase (YgeW), possibly co-expressed since the gene loci are adjacent to each other, were detected only *in vivo* (Table 2). Four small cold shock proteins, CspA in particular (Table 2) attributed to have cold shock and RNA chaperone functions [63] were strongly increased in abundance *in vivo*.

### Acid Stress

Several acid resistance stress pathways are implicated in the survival of *E. coli* strains when challenged by intracellular acidification. Our data suggest that EHEC strain 86-24 utilizes two glutamate decarboxylases to neutralize intracellular H^+^ ions and GadC for aminobutyrate export in the intestinal environment [64] (Figure 2). Due to 99% sequence identity, the decarboxylases GadA and GadB could not be discerned based on APEX_i_ data. However, their respective pI values are 5.23 and 5.29, and 2D gel profiles suggested that GadA was increased 2-fold *in vivo* vs. *in vitro*, whereas GadB was highly abundant in both experimental groups. GadC was increased 3-fold *in vivo* (Table 2). Arginine decarboxylase (AdiA) was an order of magnitude less abundant than GadA and GadB *in vivo*, implying that this enzyme was not essential to counteract an acidifying intracellular pH in bacterial cells. While the ambient pH is neutral to basic in the colon, MAF pathway activities and lactate, acetate, and formate production likely cause a pH drop in the bacterial cytoplasm supporting the notion of enhanced acid stress.

### Stress Associated with Reactive Oxygen and Nitrogen Species

**Figure 3:**
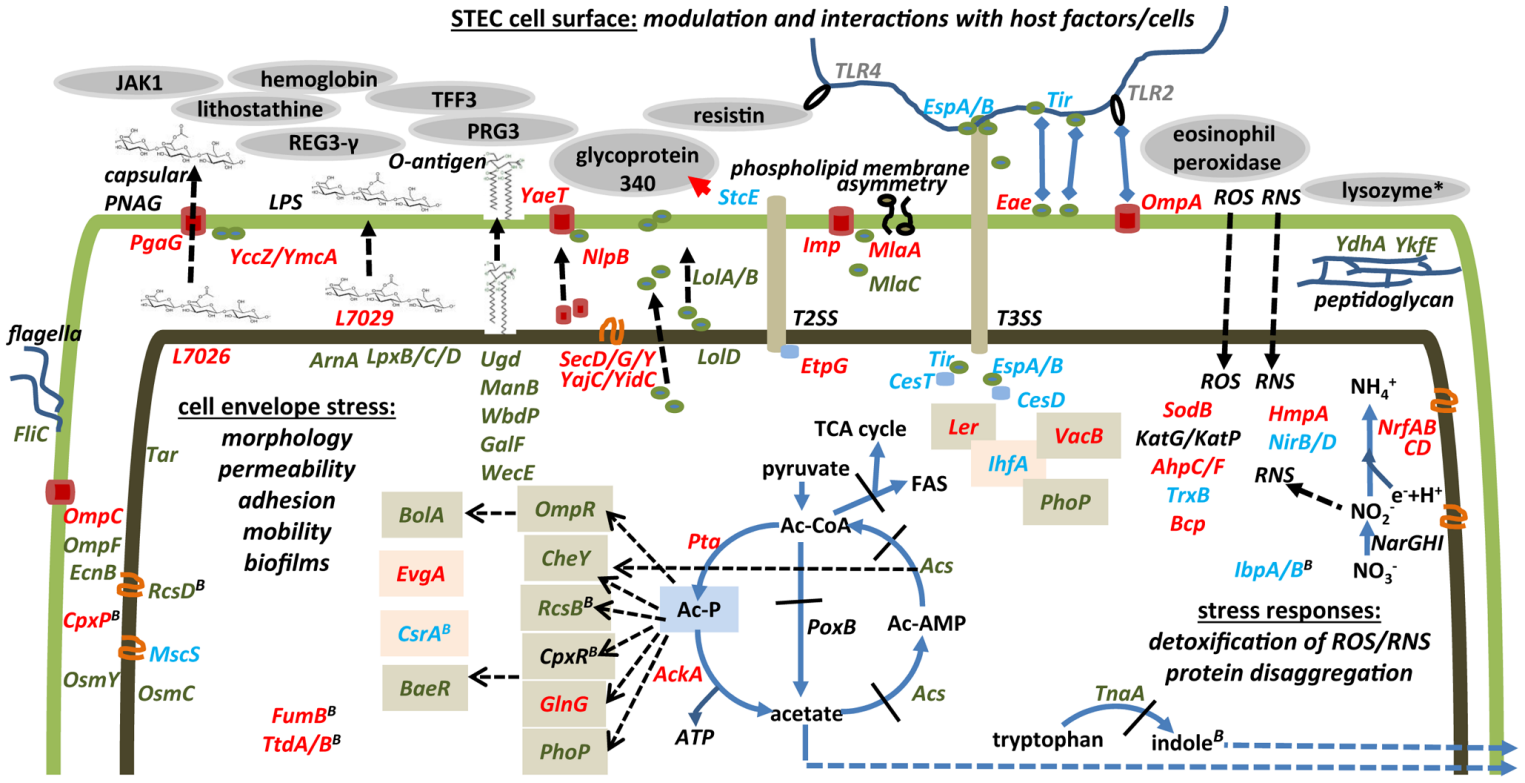
Changes in the EHEC cell envelope during effacement of the host environment and molecular responses initiated by the piglet host. *At the top of the schematic, generally from left to right: Sus scrofa* proteins identified as adhesion factors to the bacterial surface; proteins implicated in capsular poly-N-acetylglucosamine (PNAG) and lipopolysaccharide (LPS) biosynthesis; proteins implicated in β-barrel OM protein synthesis (YaeT and NlpB), lipoprotein export (LolA, LolB and LolD), OM asymmetry (Imp, MlaA and MlaC); T2SS and T3SS effectors and some of the established interactions with host cells and the extracellular matrix (StcE, EspA/B, Eae/Tir and OmpA); putative peptidoglycan-modulating proteins (YdhA and YkfE). *Below from left to right:* various extracytoplasmic stress responses; regulators of cell envelope stress and the connection to acetyl phosphate (Ac-P) signaling; tryptophan/indole biosynthesis; ROS and NOS mediated stress responses by peroxidases, catalases, dismutases and NO-detoxifying enzymes.

Enzymes removing peroxides and oxygen radicals were abundant under *in vitro* and *in vivo* conditions, including alkyl hydroperoxide reductase (AhpC), superoxide dismutase (SodB) and two catalases, KatP and KatG (Figure 3). A 2.3-fold *in vivo* increase of the oxidoreductase AhpF, which can form a complex with oxidized AhpC in the presence of an excess of NADH, was observed. AhpF binding may recycle the oxidized form of AhpC more effectively and increase AhpC activity *in vivo* [65]. SodB is an iron-dependent dismutase. Since iron starvation induces decreased *sodB* expression in many γ-proteobacteria [26], it is reasonable to conclude that EHEC cells are generally not iron-starved *in vivo*. Periplasmic SodC and cytoplasmic SodA were strongly decreased *in vivo* (Table 2), which was expected considering their transcriptional repression by Fnr and ArcA in microaerophilic environments. Higher abundances of nitric oxide dioxygenase (HmpA) and NADPH-dependent nitrite reductase (NirB/NirD) *in vivo* supported the notion that nitrite and nitric oxide (NO) stress rose considerably *in vivo* (Table 2/Figure 3). These enzymes detoxify NO [66], [67], a cytotoxic product produced by activated macrophages and neutrophils to kill invasive bacteria. In addition, NO is presumably a catalytic by-product of the *in vivo*-activated electron transporter nitrite reductase, an enzyme complex more abundant *in vivo* (e.g. NrfA, Table 1).

### Bacterial Cell Envelope Changes in the Intestinal Milieu

*E. coli* strains have complex regulatory systems modifying the cell envelope to withstand external stresses in hostile environments. In addition to OmpR, the transcription factor network coordinating the cellular responses to extracellular signals involves PhoQ/PhoP, RcsB/RcsD, RstA/RstB, OxyR, CpxA/CpxR, CheY/CheZ, LexA, BaeR/BaeS, BolA, BasR/BasS and EvgA/EvgS [59]. Nine of these regulators (PhoP, RstA, and CheY are listed in Table 2) were surprisingly decreased in abundance in intestinal EHEC cells. Complex interactions with proteins, RNAs and post-translational modifications influence the activities of these transcription factors such that expression data derived from host environmental stress does not offer conclusive insights.

For example, CpxA and CpxR form a 2-CST. CpxR was one of the more abundant extracytoplasmic stress response regulators *in vivo*. This transcription factor is involved in functions as diverse as pilus assembly, secretion, motility, chemotaxis, adherence, biofilm formation, multidrug resistance and toxin efflux [68], [69]. Two periplasmic chaperones whose respective genes are under control of CpxR were less abundant *in vivo* (PpiA and Spy). LpxD and LpxB (Table 2), two enzymes involved in lipid A core biosynthesis, a protein of unknown function (YebE) and the regulator BaeR were also decreased in abundance *in vivo*. Autokinase activity of CpxA is inhibited by CpxP, a periplasmic protein that was profiled only in intestinal EHEC cells [70]. CpxP might deactivate CpxR in bacterial cells *in vivo*, by reducing the level of phosphorylation of CpxR by CpxA. Proteomic data generally supported the notion of reduced chemotaxis and quorum sensing *in vivo*; e.g., the methyl-accepting chemotaxis protein Tar, the flagellar protein FliC, and the autoinducer (AI)-2 uptake system Lsr were lower in abundance *in vivo*. Tryptophanase (Table 1) and LuxS, enzymes involved in biosynthesis of indole and autoinducer-2 (4,5-dihydroxy-2,3-pentanedione), respectively, were strongly decreased *in vivo* vs. *in vitro*. The factor for inversion stimulation (Fis), a small DNA-binding protein which affects processes such as cell mobility, aggregation and biofilm formation via modulation of nucleoid structures [71], was quite abundant *in vivo*, but not detected *in vitro*. Regarding its regulatory capabilities, Fis overlaps with Fnr and NarL (two anaerobic respiration regulators), CpxA/CpxR, IhfB, and DksA. This is evident surveying transcription factor specificities listed in Table 2. The role of the signaling molecule acetyl phosphate (Ac-P) in EHEC cells is also interesting, as the schematic in Figure 3 reveals. Ac-P release affects the stress response transcriptional regulator network [72]. Accumulation of intracellular pyruvate resulting from lower citrate cycle metabolism and lower activities of acetyl-CoA synthetase (Acs) (Table 1) and pyruvate oxidase (PoxB) *in vivo*, combined with highly active Pta/AckA, support the intracellular accumulation of Ac-P. We hypothesize that Ac-P is an important signaling molecule in EHEC cells in the infectious process.

Five transcriptional regulators, Fis, BolA, CpxR, CpxP and RcsB, influence *E. coli* biofilm formation. Three fermentation-related enzymes (TtdA, TtdB and FumB), more abundant in EHEC *in vivo*, have also been associated with biofilms in *E. coli* [39]. Biofilm formation requires capsular polysaccharides which embed bacterial cells in an extracellular matrix structure. YgiB, YmcA, YccZ and L7026, four proteins tentatively linked to exopolysaccharide biosynthesis, were increased in EHEC cells *in vivo* and may therefore also be implicated in biofilms (Figure 3). PgaA, an export protein for poly-beta-1,6-N-acetyl-D-glucosamine identified only *in vivo* (with low abundance), is required for exopolysaccharide biosynthesis [73]. Several enzymes involved in lipopolysaccharide (LPS) biosynthesis, including the lipid A core, the O-antigen and OM phospholipid asymmetry, were also altered in abundance: ArnA and a putative lipid IVA N-acyltransferase (L7029); the O-antigen biosynthetic enzymes (Ugd, ManB, WbdP, and WecE); three proteins with functions in OM asymmetry maintenance (MlaA, MlaC, and LptD). Targeted biochemical studies may reveal whether these proteins (see also Table 2/Figure 3) have functional roles in biofilm formation and/or bacterial cell surface modulation in the host environment.

### EHEC Exposed to Infiltrating Immune Cells Following Mucosal Injury

Anticipating the ability to identify host proteins, shotgun proteomic data were queried with a *Sus scrofa* protein sequence database. We noticed that, in a subset of three *in vivo* samples, hemoglobin subunits were identified whereas, in a subset of four samples, this was not the case (here termed HB+ and HB-, respectively). Hemoglobin is a biomarker for vascular damage following EHEC infection since the protein is highly abundant only in red blood cells. Comparing the proteomic profiles of the HB+ and HB- subgroups, 138 proteins were quantitatively changed (Wilcoxon rank sum test; P<0.05). Four Ler-regulated virulence factors (StcE, Tir, EspA and Eae) and two T3SS effector chaperones (CetT and CesF) were increased in abundance at least 6-fold in the HB+ group (Table 3). The metalloprotease StcE interacts with and cleaves mucosal glycoproteins (Figure 3). EspA is a T3SS needle filament protein and essential for Tir translocation into the host cell membrane [7]. It is plausible that increased abundances of T3SS effectors and StcE facilitate bacterial translocation through M cell layers, and exposure to phagocytic cells prompts the release of Stx-2 [56]. The coincidental detection of hemoglobin supports this notion, but the lack of detection of Shiga toxin-2, supposed to be initiated during macrophage apoptosis, does not. In our experiments, EHEC cells were mostly derived from the colon where A/E lesions are reported to be less prevalent than in the ileum [56]; we can only speculate that the observed T3SS induction coincides with M cell translocation and exposure to phagocytes. Other proteins altered in abundance comparing the HB+ and HB- groups were the IhfA subunit of integration host factor which is implicated in induction of the same set of effectors controlled by Ler [14]. IhfA was increased 2.2-fold in the HB+ group. The carbon storage regulator CsrA (Table 3) showed a 3-fold increase in the HB+ group. Interestingly, a Δ*csr*A mutant strain of *S. typhimurium* was attenuated in its ability to invade HEp-2 epithelial cells [74]. Three proteins linked to intracellular survival of *S. typhimurium* in macrophages [75], [76] were highly expressed in the HB+ group, but not in the HB- group: the heat shock proteins IbpA and IbpB and the biotin synthase BioB (Table 3). These proteins may promote the intracellular survival of EHEC cells in macrophages. An intracellular life stage in those macrophages that communicate with M cells and are associated with submucosal invasion is being debated. *E. coli* O157:H7 were described to translocate into the murine ileal submucosa via extensive interactions with M cells and macrophages [56]. However, a transcriptome survey of *E. coli* O157:H7 cells invading THP-1 monocytes revealed few agreements with our proteomic data [19]. Invasion by an EPEC strain caused extensive changes in the proteome of Caco-2 intestinal cells, and this was reported to be mediated by pedestal formation and translocation of T3SS effectors [77].

Increased access to nutrients and oxygen may be attributable to the different metabolic states of HB+ compared to HB- bacterial cells, thus supporting higher cell growth rates: two carbon starvation proteins (Slp and YjiY) were decreased, whereas 21 proteins associated with aerobic energy metabolism and 11 proteins implicated in amino acid biosynthesis were increased in the HB+ group. These adaptations are not unexpected considering more nutrient- and oxygen-rich environments available to the bacteria when erythrocytes and leukocytes infiltrate the site of infection. Activation of the innate immune system in the piglets could result in the release of toxins such as reactive oxygen and nitrogen species (ROS, RNS) and antimicrobial peptides. There was little proteomic evidence for rising ROS and RNS detoxification activities mediated by SodA, SodC, AhpC, KapP, KatG and HmpA, or for the increased expression of antimicrobial efflux pumps (e.g. MdtE/MdtF) in the HB+ group. HmpA is a key enzyme neutralizing RNS. While *hmpA* induction was reported to be absent in intracellular *S. flexneri* bacteria, *S. typhimurium* revealed increased *hmpA* transcription in parallel with the onset of NO production following the invasion of macrophages [75], [78].

### Glycoprotein Adhesion to the EHEC Cell Surface

Identification of hemoglobin subunits suggested intestinal mucosal damage and enteric hemorrhage inflicted by EHEC cells. Unlike hemoglobin, most of the other identified *Sus scrofa* proteins (Table 4) were present in both *in vivo* sample subgroups. The functional roles of a few of these proteins and their tissue-specific expression were reassuring aspects as it regards the concern of unspecific binding to bacterial cells. Seven of the proteins were reported to be expressed in the intestinal or colonic epithelium of mammals. Two proteins are eosinophil products (eosinophil peroxidase, proteoglycan 3-like protein) secreted in response to microbial insults [79], [80]. Three proteins have C-type lectin domains known to have intercellular adhesive properties, including the regenerating islet-derived proteins REG3-γ and REG1-α [81], [82]. In murine models, REG3-γ is released from Paneth secretory cell granules into the intestinal lumen, unfolding antimicrobial activities against Gram-positive bacteria [81]. REG1-α (synonymous to lithostathine) is primarily secreted from acinar pancreatic cells, inhibits CaCO_3_ precipitation and also has tissue-regenerative activities. REG1-α induces bacterial aggregation *in vitro* [82]. To our knowledge for the first time, we provide evidence for adherence of these C-type lectins to Stx-producing *E. coli in vivo*. Resistin is a short cysteine-rich protein with pro-inflammatory activities that competes with LPS for binding to the Toll-like receptor 4 (TLR-4) [83]. Finally, proteoglycan 3-like protein also features a C-type lectin domain and may be engaged in pathogen pattern recognition as a co-receptor to Toll-like receptors [84] (Figure 3).

**Table 4:** Sus scrofa proteins identified from purified intestinal EHEC cells.

Protein name	HB+*	HB-*	Molecular functional roles	Tissue origin ∧	Short name
Hemoglobin subunit alpha	++	–	oxygen transport	blood	HBA1
Hemoglobin subunit beta	++	–	oxygen transport	blood	HBB1
Hemoglobin subunit epsilon	++	–	oxygen transport	blood	HBE1
Regenerating islet-derived protein 3-gamma	++	++	Innate immune defense, antibacterial - Gram(+), anti-apoptotic, PRR	GI epithelium	REG3G, C-type lectin
Lithostathine isoform 1	++	++	innate immune defense, causes E. coli aggregation, anti-apoptotic	GI epitheliumpancreas	REG1A, C-type lectin
Glycoprotein gp340	++	++	Innate immune defense, target of StcE proteases, PRR	GI epithelium	DMBT1, glycoprotein
Resistin	+	++	Pro-inflammatory, competes with bacterial LPS for TLR-4 binding	Adipocytes GI epithelium	RETN, cysteine-rich protein
Proteoglycan 3-like protein LOC 100625180	−	+	Potentially involved in cytotoxic and cytostimulatory processes	Eosinophils	PRG3, C-type lectin
Tyrosine-protein kinase JAK1	+	+	Kinase partner for the interleukin (IL)-2 receptor	Colonic epithelium	JAK1, phospho-protein
Eosinophil peroxidase	+	+	Microbicidal enzyme, implicated in inflam-matory processes and tissue remodeling	Eosinophils GI epithelium	EPX, heme+ glycoprotein
Predicted protein LOC100154068	+	+	Guanosine 5′-monophosphate oxidoreductase domain	N.K.	-
Trefoil factor 3 (intestinal)	+	+	Epithelial cell regeneration, innate immune defense	Goblet cells GI epithelium	TFF3, disulfide-rich
Predicted protein LOC100522363	+	–	Trypsin-like serine protease domain	N.K.	-

* HB+ and HB-: two in vivo EHEC groups were separated based on the identification of hemoglobin, which also correlated with high vs. low abundances of the major type III secretion system effectors; ++, +, -: the estimated protein abundances based on spectral counts (++, >8; +, <8, - none); for significance of the spectral counts, the Mascot percolator was set at q-value <0.01 and PEP value <10^−4^;

∧ Abbrev.: PRR, pattern recognition receptor; N.K., not known; GI, gastrointestinal. Details are provided in Dataset S3.

The strongest evidence for the specificity of bacterial surface binding pertains to the glycoprotein 340 (also termed GP340, deleted in malignant brain tumors 1-protein, and DMBT1). Human saliva GP340, a highly O-glycosylated and sulfated mucin contributing to the mucosal defense against pathogens, was identified as an *in vitro* substrate for StcE [12]. GP340 is also expressed in intestinal epithelial cells and responds to pro-inflammatory stimuli such as TNF-α and bacterial lipopolysaccharides (LPS). The data here provide the first *in vivo* evidence that *S. scrofa* GP340 is able to adhere to the surface of an enterohemorrhagic *E. coli* strain. The host-pathogen cross-talk results in binding of the StcE protease to GP340, likely followed by glycoprotein cleavage. These events may alter the extracellular matrix density and diminish protection of the mucosa from the formation of A/E lesions. Interestingly, Caco-2 intestinal cells infected by an EPEC strain in a T3SS-dependent manner revealed a significant GP340 abundance decrease [77]. The hypothesis that EPEC and EHEC cells send out signals manipulating intestinal secretion of critical host defense proteins, such as GP340, needs to be verified. Eosinophil peroxidase is a pro-inflammatory enzyme released during eosinophil degranulation in the gut mucosa [85]. The trefoil factor 3, a protein known to be secreted by colonic Goblet cells and involved in mucosal repair, the tyrosine-protein kinase JAK1, a signal transduction enzyme implicated in interleukin-2 and interferon-γ production, and two other proteins with unknown functions were identified (Table 4).

### Systems-level Interpretation of Proteomic Data

Investigating the EHEC proteome under human-like disease conditions is of interest to unravel molecular mechanisms of pathogenesis and identify vaccine and therapeutic drug targets. The only non-primate model with human-like pathology is the gnotobiotic piglet. What are the three main limitations of this model and our experimental approach? **First**, a normal intestinal microbiota is beneficial to the maturation of immune system and provides protection from pathogens [86]. The microbiota are absent in gnotobiotic piglets, and conventional piglets are not susceptible to EHEC disease, that seen in gnotobiotic piglets. The Stx receptor differences in pig *vs.* human may also contribute: Stx released during edema disease (in pigs) binds to receptor Gb4, Stx released during hemorrhagic colitis (in humans) binds to receptor Gb3. **Second**, bacterial cells are exposed to different host environment-induced stresses. The preferred sites for colonization, adhesion and invasion, in humans and animal models, are still under debate. The milieu acidifies in the stomach and neutralizes during passage in the small intestine, while oxygen access for respiration is gradually reduced. EHEC cells reveal tropism for the colon and ileum in humans and piglets, but the sites of adhesion and A/E lesion formation vary [87], [88]. M cells located in ileal and colonic Peyer’s Patches allow bacterial translocation in murine and *ex vivo* human models. Macrophage apoptosis triggers release of Stx in the lamina propria [19], [56]. These stages of infection cannot be separately sampled in an *in vivo* animal model. Analysis of post-mortem intestinal tissue yields averaged proteomic profiles. **Third**, host proteome profiling was limited to adherent proteins co-fractionating with *in vivo*-isolated bacterial cells. Immune responses of piglets to EHEC cell invasion were not profiled globally. We assessed proteomic evidence in support of the more invasive pathogenesis model proposed by Livrelli *et al*. [56], as opposed to the paradigm that EHEC cells largely remain in the intestinal lumen when A/E lesions are formed. It was useful to compare this data with SD1 proteome data also examined using a gnotobiotic piglet model [16], [17]. *S. dysenteriae* adapts to intracellular survival in and the spread from epithelial cells and macrophages. Unlike *S. typhimurium* [89], SD1 does not infect the host systemically with an outcome of septicemia [90].

### EHEC and SD1 Metabolize Intestinal Carbon Sources in Similar Ways

Figure 1 depicts energy metabolism pathways apparently activated in EHEC cells *in vivo* in a microaerophilic milieu. Carbon sources were discernible from those utilized during *in vitro* growth. Transporters and metabolism for lactose, galactose, mannose, N-acetylglucosamine, fructose, ascorbate, sn-glycerol-3-phosphate and C_4_ dicarboxylates were increased *in vivo*, consistent with the transcriptional modulation by 2-CSTs responsive to low oxygen concentrations [38], [41], [91], [92]. For example, DcuA and DcuB were strongly induced *in vivo*. DcuA was also increased in the SD1 proteome *in vivo* [16]. This data was not in agreement with the constitutive expression reported for *E. coli* DcuA [93]. *E. coli dcuA/dcuB* double knock-out mutants were growth-impaired when exposed to anaerobic conditions [49], suggesting this transport pathway to be a target for antibiotic inhibitor design. Short chain fatty acids also contribute to lowering the pH to 5-6 in the proximal colon [94]. EHEC is well-adapted to acid stress, and the high abundance of GadA and GadB/GadC *in vivo* revealed the pathogen’s reliance on this acid resistance system. SD1 cells also activated *in vivo* an arginine decarboxylase acid resistance system (AdiA/AdiC) and periplasmic disaggregation proteins (HdeA and HdeB) linked to acid stress [16], [17]. Stronger acid stress responses of SD1 may result from exposure to phagolysosomal conditions inside macrophages. The ability of *E. coli* O157:H7 to survive in macrophages was also examined, and transcriptome data revealed intracellular up-regulation of *hdeA, hdeD,* and *adiC* [19]. Increased transport and metabolism of fructose, lactose and galactose by EHEC and SD1 cells in the intestine may result from high lactose and fructose content of Similac Sensitive Formula, a diet the piglets were fed. The nutrient adaptations were stronger in EHEC compared to SD1 cells (a 9-fold *vs.* 4-fold change for DcuA; a 322-fold *vs.* 21-fold change for LacZ; a 24-fold *vs.* 1.7-fold for FruA/FruB). Several mixed acid fermentation enzymes and revealed stronger *in vivo* increases, citrate cycle enzymes stronger decreases in EHEC *vs.* SD1. The electron transport chains Fdo, Hyb, and Dms active at low oxygen levels were also induced more strongly *in vivo* in EHEC *vs.* SD1 cells. While strain 86-24 expressed a periplasmic nitrite reductase (deleted from the SD1 genome) *in vivo*, SD1 up-regulated a periplasmic nitrate reductase (NapA/NapB) *in vivo* [16]. Both enzymes operate at low intracellular nitrate concentrations. Proteome data for SD1 and EHEC revealed a strongly induced PHO regulon suggesting phosphate starvation *in vivo*. Evidence for both Mg and phosphate starvation in *S. typhimurium*-containing (intracellular) vacuoles [75] and in *S. flexneri* following invasion of macrophages and epithelial cells [78] was reported. The PHO regulon, apparently important in all four pathogens, may have promising targets for drug design. There was evidence for ammonia starvation in EHEC *in vivo*, considering increased expression of the AmtB-GlnK system, which controls the internal NH_4_^+^/glutamine pool and the response to ammonia shock [45]. Interestingly, these proteins were not identified in SD1 *in vivo*. To our knowledge, the GlnK-AmtB system has not been associated with increased fitness of the two pathogens during mucosal invasion processes.

Acetyl phosphate (Ac-P) may be an interesting intracellular signaling molecule in EHEC cells. Ac-P has been implicated in the functional modulation of cell envelope stress regulators which, in turn, triggers changes in cell envelope structure, motility and chemotaxis. *E. coli* Ac-P was reported to phosphorylate a few 2-CST response regulators by direct PO_4_^3−^ transfer [95]. Vilu *et al.* characterized acetate overflow metabolism in *E. coli*, when citrate cycle flux is decreased, acetyl-CoA synthetase (Acs) activity is repressed, and the enzymes Pta and AckA remain active. The disruption of the Pta-Acs node results in accumulation of Ac-P under these conditions [72]. Whereas the intestinal milieu is microaerophilic (*E. coli* growth conditions used in the aforementioned study were aerobic), proteomic data also suggested Pta-Acs node disruption in EHEC cells *in vivo* (Figure 3). Citric cycle flux and, apparently, the activities of Acs, the enzymes catalyzing subsequent fatty acid biosynthesis steps and PoxB, which converts Ac-CoA directly to acetate, were reduced *in vivo*. Increased Ac-P concentration levels may then trigger the phosphorylation of 2-CST response regulators, e.g. OmpR, PhoBR, RcsB, CheY, GlnG and CpxR [72] (Figure 3). Modulation of their DNA binding activities affects cell envelope functions [96]. RcsB, for example, is part of an *E. coli* phosphorelay system that influences biofilm formation, osmotic stress and capsular polysaccharide biosynthesis. RcsB activity is influenced by Ac-P [72]. Five proteins implicated in capsular polysaccharide synthesis were decreased in abundance in EHEC *in vivo* (Figure 3). Periplasmic OsmC, active under osmotic stress conditions and repressed transcriptionally by RcsB in *E. coli* [97], was also decreased. CpxR is another 2-CST response regulator phosphorylated by Ac-P. This post-translational modification results in the activation of expression of an adaptor protein, CpxP [98]. CpxP was identified in EHEC cells *in vivo*, but at low abundance. CpxR phosphorylation and CpxP expression *in vivo* did not result in the induction of genes implicated in cell envelope functions and believed to be under positive transcriptional control of CpxR (LpxB, LpxD, Spy, PpiA, and YebE). In fact, the proteins were decreased in abundance *in vivo* supporting the notion that 2-CSTs are part of an intricate network rather than acting alone. Finally, enzymes part of the tryptophan biosynthesis pathway including its regulator TrpR and the indole-producing tryptophanase were strongly decreased *in vivo*. Indole-based signaling was linked to biofilm formation in EHEC strain 86-24 [20], and the molecule may also modulate EHEC cell communication in the intestinal lumen. Genetic and biochemical tools are required to understand the pathogen cells’ communication patterns with each other and host defenses.

### Direct Host-pathogen Interactions

We demonstrate that host-pathogen interactions can be examined without exhaustively profiling the host cell proteome. As the example of GP340 shows, the method has biological significance. Saliva GP340 was previously identified as a substrate of metalloprotease StcE. Grys *et al.* [12] proved that StcE adheres to and cleaves this glycoprotein. The process appears to facilitate epithelial infection by EHEC [56]. The structure of the mucinase and its O-glycan chain binding site were recently characterized [99]. GP340 is a human (and apparently piglet) defense factor binding to the bacterial cell surface in a calcium-dependent manner to inhibit invasion. Other piglet proteins identified here as adhesion proteins may have similar roles in the innate immune defense: the trefoil factor 3 (TFF3) is secreted by mucin-producing goblet cells and involved in mucosal integrity [100]. Interestingly, *Citrobacter rodentium*, a model organism producing A/E lesions in mice, triggered goblet cell depletion in the colon and decreased secretion of cell-protective TFF-3 peptides [101]. Our data are the first, tentative evidence that piglet (and possibly human) TFF-3 peptides have a role in protecting Goblet cells from EHEC invasion by directly binding to the bacterial surface. Our data also provide evidence of resistin binding to the pathogen’s surface. Resistin was determined to compete with LPS for TLR-4 binding in THP-1 monocyte cell cultures [83]. We hypothesize that secreted pig resistin has similar danger signaling and immunomodulatory roles when bacterial cells come into contact with the luminal surface of ileum and colon. Three C-type lectins were identified. The bactericidal lectin REG3-γ is released from gut mucosal cells, reported to bind to Gram (+) bacteria and recognize peptidoglycan structures [81]. Our study suggests that pig REG3-γ effectively binds to EHEC cell surfaces, but its bactericidal activity needs to be verified. The proteoglycan 3-like protein PRG3 and lithostathine, an abundant pancreatic juice protein, have not been associated with innate immunity to date. PRG3 may have a functional role similar to that of REG3-γ. An intriguing role as a protein causing aggregation of *E. coli* cells was attributed to lithostathine, but not verified *in vivo* [82]. Lithostathine adheres to the EHEC surface, but a bacterial aggregation function and interference with mucosal wall damage needs to be examined. C-type lectins are a diverse family of proteins implicated in pattern recognition, TLR surface receptor cross-linking, and bacterial opsonization. These proteins modulate innate and adaptive immune responses [84], [102]. Interactions at the cell surface (Figure 3) may occur to a lesser degree when commensal gut microbes are present in conventional piglets. The competing bacteria provide a crucial line of defense against infection with pathogens and inflammation [94].

Our data provide tentative support for the murine EHEC pathogenesis model proposed by Livrelli *et al.*, where invasion of M-cells is followed by bacterial uptake by and temporary survival in macrophages that underlie M-cells [56]. Our proteomic data support two bacterial sample subgroups, one with a more aerobic metabolism and hemoglobin binding (HB+), the other with a less aerobic metabolism and no hemoglobin (HB-). Identification of hemoglobin suggested vascular damage and a more invasive stage of the bacteria where exposure to more oxygen re-activates aerobic respiration and NADH oxidase activity of infiltrating inflammatory cells mediates the respiratory burst [103]. In the HB+ group, we noticed increased expression of three proteins considered particularly important for intracellular survival in macrophages. Biotin synthase B expression was proposed to be essential for *S. typhimurium* replication inside biotin-starved macrophages [76]. The chaperones IbpA and IbpB were abundance-increased in *S. typhimurium* upon macrophage entry and functionally associated with stress responses to oxidative burst. We also observed high abundance of SodB and HmpA in the HB+ group, but not the expression increases reported for the two protein orthologs of *S. typhimurium* residing in macrophages [75]. The catalytic activities of SodB and HmpA, which detoxify ROS and RNS, respectively, are important for intracellular survival of *S. flexneri*, *S. typhimurium* and likely also *E. coli* O157:H7 strains [19], [75], [104]. An *E. coli* O157:H7 transcriptome analysis revealed low THP-1 macrophage invasion rates 24h post-infection, but in macrophages wherein bacteria were detected clusters of *E. coli* cells had formed suggesting bacterial replication. This study by Poirier *et al.* [19] did not support strong T3SS effector abundance increases during the time course of infection; our *in vivo* data were indicative of increased effector expression (Tir, Eae, EspA, and StcE) and their regulators (Ler and IhfA) in the more invasive stage (HB+ group). In contrast, expression of Shiga toxin (Stx2b) and iron transporters (Fit, Chu) were found to be induced in the course of infection [19], but not observed in the comparison of the HB+ *vs.* HB- proteome profiles. Finally, the pathogen patterns recognition molecules were not identified specifically in one of the two EHEC sample subgroups. Their binding to bacterial cells is expected to occur in the environment of the intestinal lumen.

### Concluding Remarks

A comprehensive proteomic analysis comparing EHEC cells derived from cell cultures and following bacterial collection from the gut lumen of infected gnotobiotic piglets unraveled multiple biochemical pathways including microaerophilic respiration, carbon utilization, acid resistance, and responses to several stresses (RNS, phosphate, ammonium and NADPH starvation) that were activated *in vivo*. There was evidence for extensive adaptations of the bacterial envelope to the intestinal milieu, influenced by a dedicated transcription factor network and acetyl phosphate signaling. For the first time, a set of piglet adhesion factors with likely functions in pathogen recognition and innate immune system activation was identified. The study resulted in the identification of proteins potentially useful for targeted drug design and set the stage for a significant systems biology role to understand interactions of pathogens with their mammalian hosts in an environment reflecting the normal infectious process.

## Supporting Information

Figure S1 **Differential 2D gel display of proteins from lysates of EHEC cells isolated from cell culture versus piglet intestines.** Gel image B368. This 2D gel image visualizes the proteome of EHEC strain 86-24 cells grown to the stationary phase in LB media (*in vitro*) with no exposure to the piglet host organism. Gel image B276. This gel visualizes the proteome of bacterial cells isolated from piglet intestinal lavage after infection with EHEC bacteria, observation of symptoms such as bloody diarrhea and fever, and post-mortem recovery of colon and caecum sections of the intestines (*in vivo*). The gel images range from the Mr 200 kDa (top) to 10 kDa (bottom) and the pI of 4.5 (left to the pI of 7 (right). The protein spots marked in RED are those increased in abundance in the denoted gel (sample) group, those in BLUE were decreased in the denoted gel (sample group). The spot numbers are equivalent to those in the column “AO” of Supplementary Dataset S1. EHEC cells were suspended in a 2D gel rehydration buffer (GR lysis buffer). This solution contained 8 M urea, 2 M thiourea, 4% (w/v) CHAPS, 18 mM DTT and 0.5% (v/v) Bio-Lyte pH 3-10 carrier ampholytes). Samples were frozen at −80°C until further use. On the day before 2D gel separation, bacterial lysates were thawed, incubated at 20°C for 30 min and vortexed intermittently to complete protein solubilization. Whole cell lysates were centrifuged at 16,100×g for 30 min, and supernatants were subjected to protein quantification. Supernatant samples were subjected to 2D gel electrophoresis in batches of 12 gels using procedures described in the main text. Briefly, 1^st^ dimension protein analysis in 24 cm immobilized linear pH gradient strips (pH range 4–7; GE Healthcare) included gel rehydration loading of samples with ∼150 µg protein and electrophoresis for ∼60,000 Vh. Following reduction and alkylation steps, re-equilibrated strips were applied to 2^nd^ dimension SDS-PAGE slab gel electrophoresis (25×19.5×0.15 cm; 8–18%T) for ∼1,800 Vh. Gels were fixed, stained with Coomassie Brilliant Blue G250 (CBB), de-stained, subjected to gel image analysis (data acquisition as 16 bit TIFF images) and imported into the software tool Proteomweaver v4 (Bio-Rad, Hercules, CA). The differential display analysis was performed comparing at least six gel replicate experiments (images) for the *in vitro* and *in vivo* EHEC groups. The software-based quantitative and statistical analysis method is described in the main text.
(PDF)

Dataset S1 **Protein abundance differences comparing EHEC strain 86–24 cells cultured** ***in vitro*** **versus EHEC strain 86–24 cells isolated from the intestine of infected piglets.** This Table includes data from a shotgun proteomics analysis computing protein values using the APEX Quantitative Proteomics Tool and from a differential 2D gel display analysis computing protein values in the gel image analysis software Proteomweaver v4. Detailed legends are provided in the 1^st^ worksheet of the dataset.
(XLSX)

Dataset S2 **Protein abundance differences comparing EHEC strain 86–24 cells isolated from a piglet subgroup positive for hemoglobin versus a piglet subgroup negative for hemoglobin.** This Table includes data from a shotgun proteomics analysis computing protein values using the APEX Quantitative Proteomics Tool. Detailed legends are provided in the 1^st^ worksheet of the dataset.
(XLSX)

Dataset S3 **Protein identifications searching the LC-MS/MS data from** ***in vivo*** **and** ***in vitro*** **EHEC samples with the** ***Sus scrofa*** **protein sequence database (RefSeq in NCBI).** Detailed legends are provided in the 1^st^ worksheet of the dataset.
(XLSX)
