# Supplementary material for: Proteomic View of Interactions of Shiga Toxin-Producing Escherichia coli with the Intestinal Environment in Gnotobiotic Piglets
Source: PLoS One. 2013 Jun 19;8(6):e66462. doi: 10.1371/journal.pone.0066462 (PMC3686733; doi:10.1371/journal.pone.0066462)
Supplement: Figure S1 — Differential 2D gel display of proteins from lysates of EHEC cells isolated from cell culture versus piglet intestines. Gel image B368. This 2D gel image visualizes the proteome of EHEC strain 86-24 cells grown to the stationary phase in LB media (in vitro) with no exposure to the piglet host organism. Gel image B276. This gel visualizes the proteome of bacterial cells isolated from piglet intestinal lavage after infection with EHEC bacteria, observation of symptoms such as bloody diarrhea and fever, and post-mortem recovery of colon and caecum sections of the intestines (in vivo). The gel images range from the Mr 200 kDa (top) to 10 kDa (bottom) and the pI of 4.5 (left to the pI of 7 (right). The protein spots marked in RED are those increased in abundance in the denoted gel (sample) group, those in BLUE were decreased in the denoted gel (sample group). The spot numbers are equivalent to those in the column “AO” of Supplementary Dataset S1. EHEC cells were suspended in a 2D gel rehydration buffer (GR lysis buffer). This solution contained 8 M urea, 2 M thiourea, 4% (w/v) CHAPS, 18 mM DTT and 0.5% (v/v) Bio-Lyte pH 3-10 carrier ampholytes). Samples were frozen at −80°C until further use. On the day before 2D gel separation, bacterial lysates were thawed, incubated at 20°C for 30 min and vortexed intermittently to complete protein solubilization. Whole cell lysates were centrifuged at 16,100×g for 30 min, and supernatants were subjected to protein quantification. Supernatant samples were subjected to 2D gel electrophoresis in batches of 12 gels using procedures described in the main text. Briefly, 1st dimension protein analysis in 24 cm immobilized linear pH gradient strips (pH range 4–7; GE Healthcare) included gel rehydration loading of samples with ∼150 µg protein and electrophoresis for ∼60,000 Vh. Following reduction and alkylation steps, re-equilibrated strips were applied to 2nd dimension SDS-PAGE slab gel electrophoresis (25×19.5×0.15 cm; 8–18%T) for ∼1,800 V [file pone.0066462.s001.pdf]

B368 - in vitro (in vitro vs. in vivo)

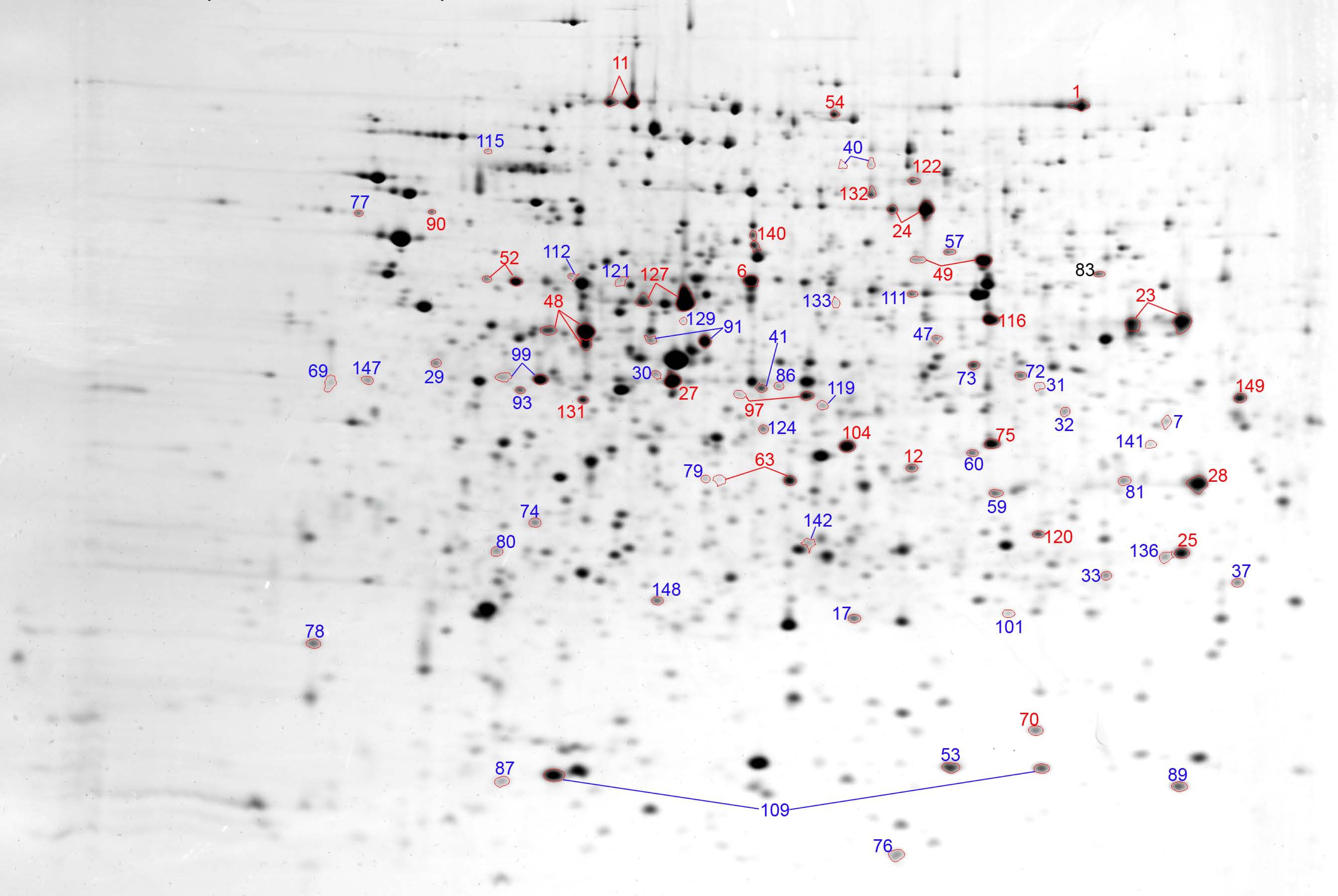

B276 - in vivo (in vitro vs. in vivo)

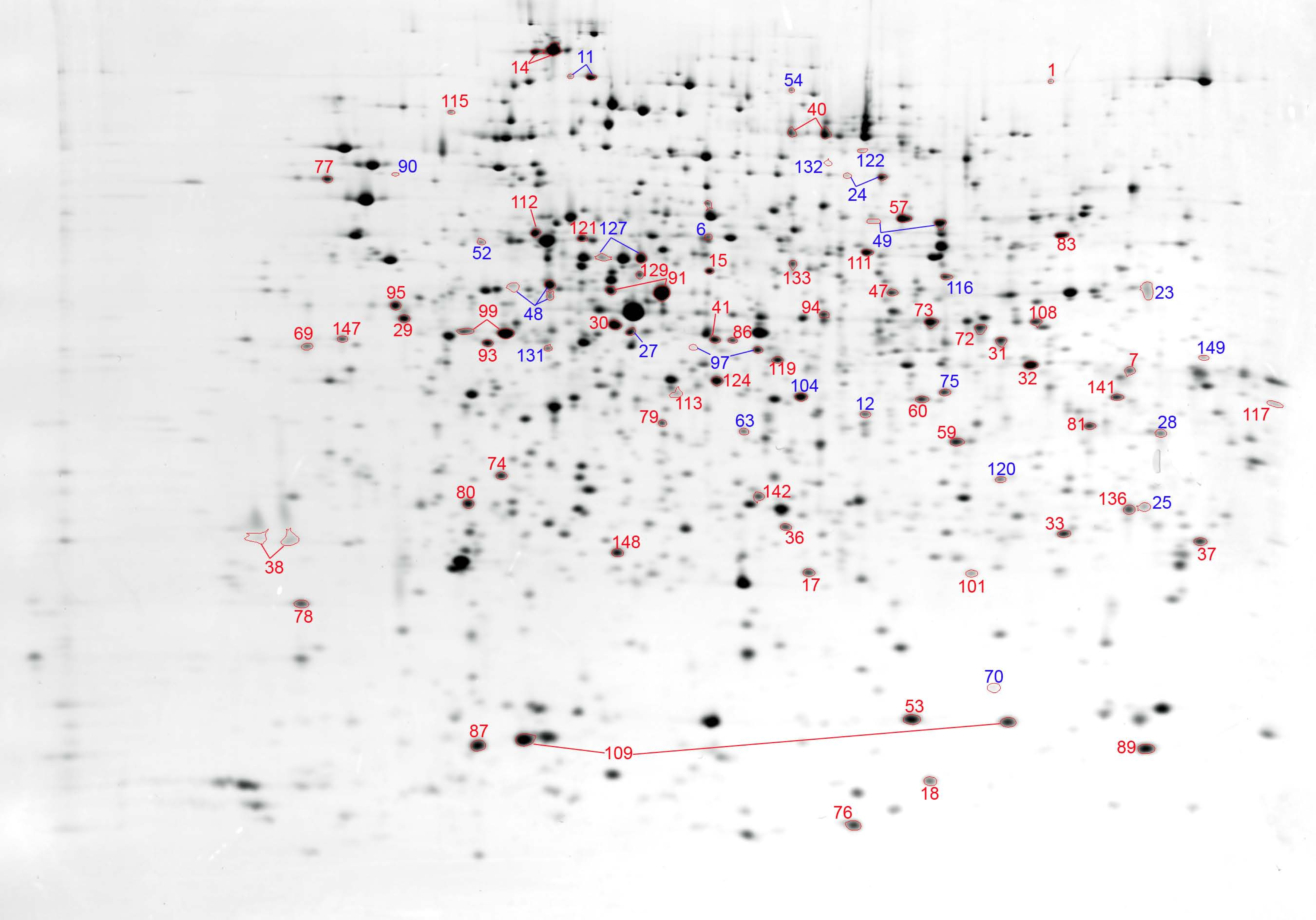

Legend for Figure S1.

Gel image B368. This 2D gel image visualizes the proteome of EHEC strain 86-24 cells grown to the stationary phase in LB media (***in vitro***) with no exposure to the piglet host organism. Gel image B276. This gel visualizes the proteome of bacterial cells isolated from piglet intestinal lavage after infection with EHEC bacteria, observation of symptoms such as bloody diarrhea and fever, and post-mortem recovery of colon and caecum sections of the intestines (***in vivo***). The gel images range from the Mr 200 kDa (top) to 10 kDa (bottom) and the pI of 4.5 (left to the pI of 7 (right). The protein spots marked in RED are those increased in abundance in the denoted gel (sample) group, those in BLUE were decreased in the denoted gel (sample group). The spot numbers are equivalent to those in the column “AO” of Supplementary Dataset S1.

EHEC cells were suspended in a 2D gel rehydration buffer (GR lysis buffer). This solution contained 8 M urea, 2 M thiourea, 4% (w/v) CHAPS, 18 mM DTT and 0.5% (v/v) Bio-Lyte pH 3-10 carrier ampholytes). Samples were frozen at -80°C until further use. On the day before 2D gel separation, bacterial lysates were thawed, incubated at 20°C for 30 min and vortexed intermittently to complete protein solubilization. Whole cell lysates were centrifuged at 16,100 × g for 30 min, and supernatants were subjected to protein quantification. Supernatant samples were subjected to 2D gel electrophoresis in batches of 12 gels using procedures described in the main text. Briefly, 1st dimension protein analysis in 24 cm immobilized linear pH gradient strips (pH range 4-7; GE Healthcare) included gel rehydration loading of samples with ~150 µg protein and electrophoresis for ~60,000 Vh. Following reduction and alkylation steps, re-equilibrated strips were applied to 2nd dimension SDS-PAGE slab gel electrophoresis (25 x 19.5 x 0.15 cm; 8-18%T) for ~1,800 Vh. Gels were fixed, stained with Coomassie Brilliant Blue G250 (CBB), de-stained, subjected to gel image analysis (data acquisition as 16 bit TIFF images) and imported into the software tool Proteomweaver v4 (Bio-Rad, Hercules, CA). The differential display analysis was performed comparing at least six gel replicate experiments (images) for the *in vitro* and *in vivo* EHEC groups. The software-based quantitative and statistical analysis method is described in the main text.
